# RuBisCO depletion improved proteome coverage of cold responsive S-nitrosylated targets in Brassica juncea

**DOI:** 10.3389/fpls.2013.00342

**Published:** 2013-09-02

**Authors:** Ankita Sehrawat, Jasmeet K. Abat, Renu Deswal

**Affiliations:** Molecular Plant Physiology and Proteomics Laboratory, Department of Botany, University of DelhiDelhi, India

**Keywords:** S-nitrosylation, thiol pool, nitric oxide signaling, cold stress

## Abstract

Although in the last few years good number of S-nitrosylated proteins are identified but information on endogenous targets is still limiting. Therefore, an attempt is made to decipher NO signaling in cold treated *Brassica juncea* seedlings. Treatment of seedlings with substrate, cofactor and inhibitor of Nitric-oxide synthase and nitrate reductase (NR), indicated NR mediated NO biosynthesis in cold. Analysis of the *in vivo* thiols showed depletion of low molecular weight thiols and enhancement of available protein thiols, suggesting redox changes. To have a detailed view, S-nitrosylation analysis was done using biotin switch technique (BST) and avidin-affinity chromatography. Ribulose-1,5-bisphosphate carboxylase/oxygenase (RuBisCO) is S-nitrosylated and therefore, is identified as target repeatedly due to its abundance. It also competes out low abundant proteins which are important NO signaling components. Therefore, RuBisCO was removed (over 80%) using immunoaffinity purification. Purified S-nitrosylated RuBisCO depleted proteins were resolved on 2-D gel as 110 spots, including 13 new, which were absent in the crude S-nitrosoproteome. These were identified by nLC-MS/MS as thioredoxin, fructose biphosphate aldolase class I, myrosinase, salt responsive proteins, peptidyl-prolyl cis-trans isomerase and malate dehydrogenase. Cold showed differential S-nitrosylation of 15 spots, enhanced superoxide dismutase activity (via S-nitrosylation) and promoted the detoxification of superoxide radicals. Increased S-nitrosylation of glyceraldehyde-3-phosphate dehydrogenase sedoheptulose-biphosphatase, and fructose biphosphate aldolase, indicated regulation of Calvin cycle by S-nitrosylation. The results showed that RuBisCO depletion improved proteome coverage and provided clues for NO signaling in cold.

## Introduction

Research in the last two decades has proved beyond doubt, the versatility of nitric oxide (NO) as an important signaling molecule in plants. It regulates numerous biological processes (Besson-Bard et al., 2008). Despite this, relatively little is known about its downstream signaling pathways. NO predominantly manifests its effects by post-translational modifications (PTMs) like S-nitrosylation, glutathionylation and tyrosine nitration. S-nitrosylation is the most investigated PTM, which regulates the physiological processes (Kovacs and Lindermayr, 2013). Once the physiological relevance of S-nitrosylation was established, the next phase of research focused on the identification of the putative S-nitrosylated targets, to establish the signaling mechanism.

S-nitrosylated proteins were identified from *Arabidopsis thaliana* (Lindermayr et al., 2005), *Kalanchoe pinnata* (Abat et al., 2008), *Brassica juncea* (Abat and Deswal, 2009), *Solanum tuberosum* (Kato et al., 2012), *Oryza sativa* (Lin et al., 2012) and *Pisum sativum* (Camejo et al., 2013). S-nitrosoproteome analysis is mostly done using NO donor because of the low concentration of endogenous S-nitrosothiols (SNOs). It is mandatory to identify and validate the endogenously S-nitrosylated proteins not only to confirm the targets identified using donors but also to understand their physiological relevance.

For the identification of S-nitrosylated proteins, biotin switch technique (BST, Jaffrey and Snyder, 2001) is used. It involves the selective reduction of the SNOs by ascorbate, their substitution with biotin and their purification by avidin-affinity chromatography. A major drawback of this procedure is the masking of the low abundant S-nitrosylated proteins by the abundant ones like Ribulose-1,5-bisphosphate carboxylase/oxygenase (RuBisCO, Abat and Deswal, 2009), RuBisCO activase (Tanou et al., 2012), glyceraldehydes-3-phosphate dehydrogenase (GAPDH, Maldonado-Alconada et al., 2010) and heat shock proteins (Maldonado-Alconada et al., 2010). These proteins saturate the avidin column and compete out the low abundant S-nitrosylated proteins. Besides hindering the detection of the low abundant targets, these also waste precious effort and time during MS identification. This prompted us to remove RuBisCO to improve the chances of getting the regulatory S-nitrosylated targets.

Recently, a NO-cold crosstalk was proposed at genes, lipid and protein level, but the regulatory mechanisms involved are still elusive (Sehrawat et al., 2013). Therefore, to get a better understanding of these signaling pathways, identification of the regulatory targets is essential. Previously, cold mediated inhibition of RuBisCO by S-nitrosylation was shown (Abat and Deswal, 2009), on the similar lines other signaling targets need to be functionaly validated. Therefore, the aim of this study was to demonstrate if the repertoire of cold responsive S-nitrosoproteome could be enriched by removing RuBisCO. Furthermore, the effect of S-nitrosylation on the superoxide dismtase (SOD) activity, a cold responsive S-nitrosylated target (identified in this study), was validated to understand its regulation by NO. In addition, to establish the NO signaling in cold, NO production and modulation of the *in vivo* thiol pool by NO was measured.

## Materials and methods

### Plant material and growth conditions

*Brassica juncea* var. pusa jaikisan seeds were obtained from The Indian Agricultural Research Institute, New Delhi, India. Seeds were surface sterilized with 70% ethanol for 10 min and soaked overnight in double distilled water. Seeds were placed in the wet germination paper rolls and kept overnight in dark. These were transfered to a growth chamber at 25°C under white fluorescent light (270 μmol/m^2^/s, 16 h light/8 h dark) for 7 days.

### Cold stress, SNP (sodium nitroprusside) and cPTIO (2-pHENYL-4,4,5,5-TETREMETHYL-IMIDAZOLINE-1-OXYL-3-OXIDE) treatment

For cold stress, 7 days old seedlings were kept in a cold chamber at 4°C for 2–96 h under the same conditions as mentioned in the above section. Control seedlings were kept at 25°C. Seedlings were treated with SNP (a NO donor, 50, 100, 250 μM) or cPTIO (a NO scavenger, 100 μM). Following the treatment, the seedlings were rinsed with the double distilled water and blotted onto a filter paper and were immediately frozen in the liquid nitrogen.

### Nitric oxide measurement

NO was measured using the NO measuring system (inNO, Innovative Instruments Inc.) following manufacturer's instructions. inNO consist of a nitric oxide meter, a sensor and a data acquisition system which measure free NO in the sample. NO measurement experiments were performed following (Modolo et al., 2005). In brief, seedlings (1:1, w/v) were homogenized in sodium phosphate buffer (100 mM, pH 7.4). Homogenate was centrifuged at 10,000 g (Beckman Coulter, Allegra 64R) for 10 min at 4°C. The supernatant was passed through two layer of cheese cloth and incubated for 1 h at 25°C with L-arginine (1 mM), NADPH (1 mM) with L-arginine (1 mM), NG-nitro-L-arginine methyl ester (1 mM, L-NAME), sodium nitrite (1 mM), NADH (1 mM) with sodium nitrite (1 mM) and sodium tungstate (1 mM) in different sets. NO was expressed as nM/ min /g FW.

### Thiol pool measurement

The thiol pool was measured following (Ivanov and Kerchev, 2007) with some modifications like the control and cold (6 h) treated seedlings were homogenized in the extraction buffer in 1:1 (w/v) ratio. Additionally, the pellets, P1 and P2 were re-suspended in a detergent solution using a sonicator (Ultrasonic Vibra cell), for the better solubilization of the thiols. A separate set of glassware was used to prevent any contamination with the metal ions. In addition, the entire experiment was performed at low temperature as the oxidation of thiols is temperature dependent. The results were expressed as μ moles -SH/g FW. Different fractions for analysis were: pellet bound thiols, pellet obtained after the first centrifugation (P1); available thiols, supernatant 1- supernatant 2 (S1–S2); low molecular weight thiols, S2; total protein thiols, pellet obtained after trichloroacetic acid (TCA) precipitation (P2); buried thiols, total protein thiols—available thiols; total thiols, total protein thiols + low molecular weight thiols + pellet bound thiols.

### RuBisCo depletion by PEG precipitation and immunoaffinity purification

For RuBisCO depletion, the seedlings were extracted (1:3, w/v) in 20 mM Tris-HCl (pH 7.0) containing 20% glycerol and 5 mM PMSF. The homogenate was centrifuged at 12,000 rpm for 20 min at 4°C. Protein was estimated by Bradford assay (Bradford, 1976). The supernatant was used for the RuBisCO depletion experiments. For PEG precipitation, PEG 4000 [60% (w/v)] was added to the supernatant (5–15%) with stirring. After 30 min of stirring at 4°C, the extract was centrifuged at 16,000 g for 45 min. The pellet and the supernatant thus obtained were dissolved in the sample buffer and loaded on a 12% SDS-PAGE gel.

For the immunoaffinity purification, Seppro IgY RuBisCO Spin Column kit (Sigma–Aldrich) was used following the manufacturer's instructions. Briefly, the column was washed thrice before use with 500 μL tris buffered saline (TBS, 1 mM Tris-HCl, 150 mM NaCl, pH 7.4) to remove the suspension buffer. Immuno-capture of RuBisCO was performed by incubating the supernatant (90 μg protein) with the matrix for 15 min at 25°C with gentle shaking. After 15 min, the flow through was collected by centrifugation at 2000 rpm for 30 s. Unbound protein were removed by washing with TBS. Elution was done with the stripping buffer (100 mM glycine-HCl, pH 2.5) and the fractions were immediately neutralized with 1M Tris-HCl, pH 8.0.

### Detection and the purification of the S-nitrosylated proteins

The S-nitrosylated proteins were detected and purified from RuBisCO depleted fractions by BST and neutravidin-agarose column chromatography following Abat and Deswal (2009) except that the GSNO, GSH, and DTT were removed using micro Bio-Spin 6 columns (Bio-Rad). For the purification of the cold modulated S-nitrosylated proteins, extraction and purification of RuBisCO depleted proteins was performed in dark to prevent the light induced degradation of SNOs. Separate Seppro columns were used for the control and the cold treated samples to avoid cross contamination. Stress induced S-nitrosylation was analyzed from the RuBisCO depleted fractions (5 mg) obtained from cold (6 h, 4°C) treated seedlings as mentioned above. S-nitrosylated proteins were resolved on 1-D and 2-D gels. Experiment was repeated with three biological replicates.

### Two-dimensional electrophoresis

Two dimensional electrophoresis was performed following Abat and Deswal (2009) with minor modifications. In the lysis buffer, 0.75% ampholytes was used to increase the solubilization of proteins. The gels were stained with the MS compatible silver staining as described by Yan et al. (2000). Three biological replicates were performed for each sample.

### Image acquisition, data analysis and protein identification by nLC-MS/MS

The gels were scanned using Alpha Imager (Alpha Innotech, Corporation). ImageMaster 2-D Platinum software (version 6.0, GE Healthcare, Sweden) was used for the spot detection in the 2-D gels. Protein spot pattern from the gels of three independent biological replicates were used to create a master gel in the first level match set. The gels were normalized in the percentage spot volume mode to reduce the differences in the protein loading and gel staining. This was followed by the formation of a second level match set where master gel of different samples was compared. Intensity of each spot is defined as the sum of the intensities of the pixels constituting that spot and is represented in the spot volume. Students's *t*-test (*p* < 0.05) was applied to determine any significant quantitative change.

For the MS identification, polypeptides/spots were manually excised from silver stained 1-D or 2-D gel. Identification was done at Proteomics International by Electrospray mass spectrometry on a 4000 Q TRAP mass spectrometer (Applied Biosystems). Utimate 3000 nanoflow LC system (Dionex, Bannockburn, IL, USA) was used for sample introduction as described in Bringans et al. (2008). The peak list obtained was submitted to the MASCOT search engine (http://www.matrixsciences.com) and was searched against the NCBInr (20130407 24070523 sequences; 8281664780 residues) in Viridiplantae. The search parameters were same as described in (Abat and Deswal, 2009) with peptide mass tolerance—±0.8 Da, and instrument type—ESI-QUAD-TOF. The significant hits identified by MASCOT probability analysis (*p* < 0.05) with mowse score 50 and above were selected. The unidentified/hypothetical proteins were subjected to BLASTP search against the NCBInr protein database to assign function to the unnamed or unknown proteins.

### Superoxide dismutase and fructose bisphosphate aldolase activity assay

For the enzyme assays, the seedlings were extracted in the HEN buffer (250 mM Hepes-NaOH pH 7.7, 1 mM EDTA, 0.1 mM Neocuproine, pH 7.4, 1:3, w/v) and the homogenate was centrifuged at 14,000 g for 25 min at 4°C. The supernatant was passed through two layers of the cheese cloth and was incubated without or with GSNO (100-500 μM) or GSH (250 μM) in the dark for 20 min at 25°C. For the DTT treatment, after incubation with GSNO (100 μM), the samples were incubated with DTT (10 mM) in dark for 40 min. GSNO, GSH and DTT were removed using Micro Bio-Spin 6 columns (Bio-Rad).

The total SOD (EC 1.15.1.1) activity was assayed by monitoring the inhibition of photochemical reduction of nitroblue tetrazolium [NBT, (Beyer and Fridovich, 1987)]. The reaction mixture (1.5 ml) contained 33 μg of protein extract, phosphate buffer (50 mM, pH 7.8), EDTA (0.1 μM), methionine (13 mM), NBT (75 μM) and riboflavin (2 μM). One unit of SOD activity is defined as the amount of enzyme which causes 50% inhibition in the NBT reduction. Optical density was recorded at 560 nm using a UV-spectrophotometer (Beckman Coulter, DU-730).

Fructose bisphosphate aldolase activity assay was done based on Boyer's modification of hydrazine assay following Richards and Rutter (1961). This assay is based on reaction of 3-phosphoglyceraldehyde (product of fructose bisphosphate aldolase) with hydrazine to form hydrazone which absorbs at 240 nm. One unit of enzyme is defined as a change in absorbance/min at 25°C. The assay mixture contained 0.012 M fructose-1, 6 bisphosphate, 0.1 mM EDTA containing 3.5 mM hydrazine sulfate. After recording the absorbance 240 nm for 10 min, the enzyme (25 μl) was added and the absorbance was recorded further for 10 min.

### Statistical analysis

Intensity of polypeptide in SDS-PAGE gels was quantified by densitometric scanning (AlphaImager software, Alpha Innotech Corporation) with three repeats. The data shown in the NO measurement, thiol pool analysis and the enzymatic assay represents mean ± SD from three independent experiments performed in triplicates and significant differences were calculated by Student's *t*-test with *p* ≤ 0.05.

## Results

### Cold stress enhanced endogenous nitric oxide production and modified the *in vivo* thiols

**Figure 1:**
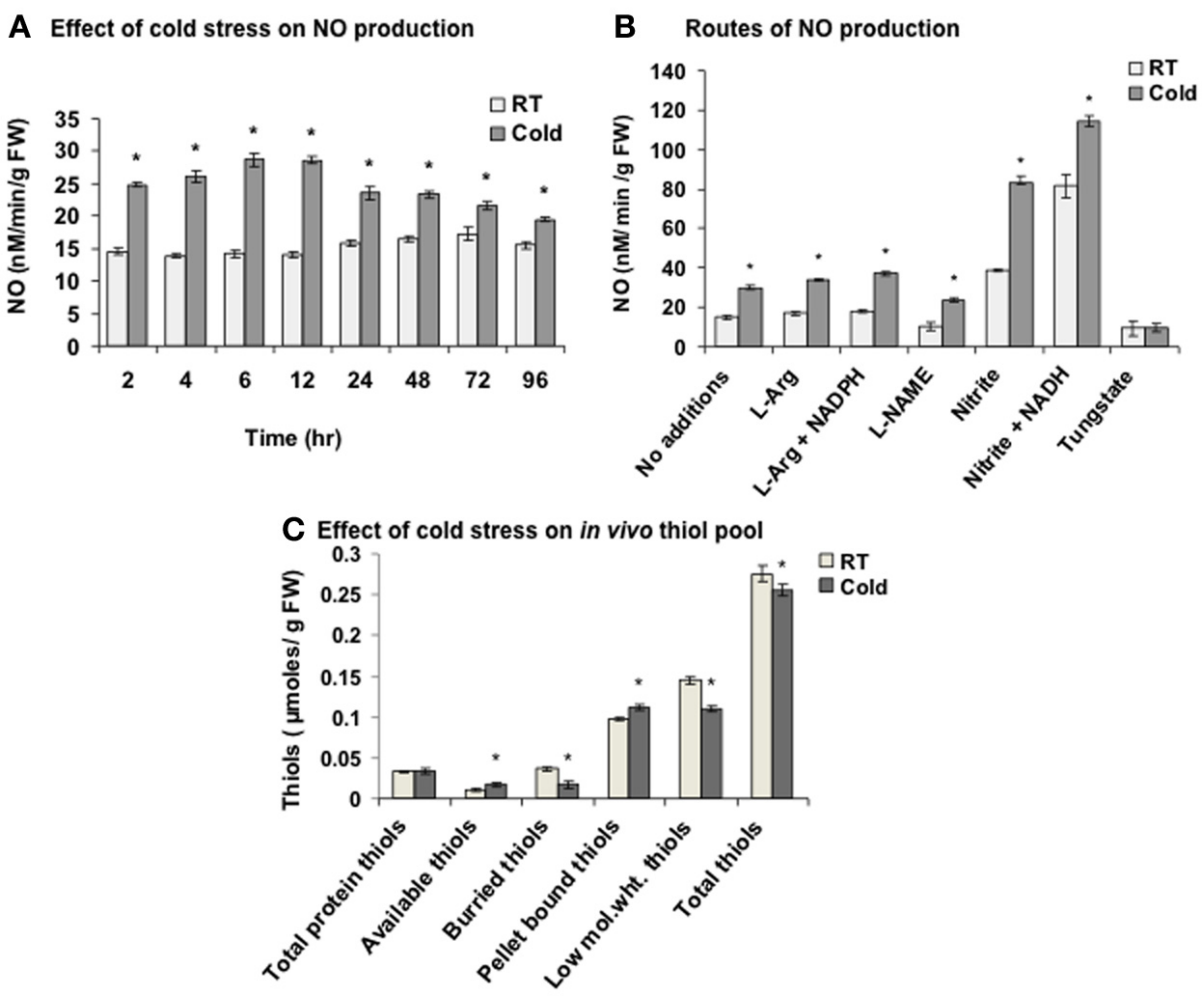
**Nitric oxide (NO) and *in vivo* thiol pool measurement in cold. (A)** NO production measured using NO measuring system after the cold treatment (2–96 h) to the seedlings. **(B)** NO production in the extract from control (room temperature, RT) and cold (4°C) treated seedlings with L-arginine (L-Arg, 1 mM) alone or with NADPH (1 mM), L-NAME (1 mM), nitrite (1 mM) alone or with NADH (1 mM) and tungstate (1 mM). **(C)** Alterations in the thiol pool in cold (6 h) treated seedlings. Low mol. wt. thiols; low molecular weight thiols. Results represent mean ± SD from three independent experiments performed in triplicates. Asterisk (*) indicates significant differences between control and cold with *p* = 0.05 calculated using Student's *t*-test.

NO content was measured in the cold (4°C) treated seedlings using NO measuring system. The sensing element of the iNO sensor has a NO selective permeable membrane. Cold stress led to NO evolution right from 2 h with maximum NO accumulation (2 fold) at 6 h (Figure 1A). In control (25°C, RT), negligible increase (at 72 h) was observed. Nitric oxide synthase (NOS)-like enzyme and nitrate reductase (NR) are the two key enzymes responsible for the NO production in plants. Addition of L-arginine (1 mM, substrate of NOS-like enzyme) alone or with NADPH (1 mM, cofactor of NOS) showed 1.1 and 1.23 fold increase respectively in NO in cold (6 h), while L-NAME (1 mM, an inhibitor of NOS) brought it back to the basal level (Figure 1B). In contrast, nitrite (1 mM, a substrate for NR) alone or along with NADH (1 mM, a cofactor of NR) increased the NO production by 2.78 and 3.72 fold respectively indicating primarily NR mediated NO production in cold. Higher NO production than the *in vivo* NO generating capacity of the plant, could be due to the higher concentrations of the substrate and cofactors being provided from the outside. A decrease in NO to the basal level by tungstate (1 mM, an inhibitor of NR) in cold confirmed the results. The control (RT) sets showed a similar trend.

As NO modulates the cellular thiols, these were quantified in cold stress. Thiols are broadly categorized into protein-based (high molecular weight), non-protein based (low molecular weight) and pellet bound thiols. Protein-based thiols are further categorized as available and buried thiols. Low molecular weight thiols include GSH and free cysteines. Pellet bound thiols are the thiols present in the broken organelles and cell membranes. Cold stress increased available thiols and pellet bound thiols by 54.5% and 14.2% respectively, while decreased the buried thiols and low molecular weight thiols by 53.8% and 24.1% respectively (Figure 1C). Overall, 7.2% decrease in the total thiols was observed in cold. One of the reason for this decrease could be the reaction of cold induced NO with low molecular weight thiols like GSH to yield GSNO leading to S-nitrosylation.

S-nitrosylation analysis of the regulatory targets is challenging due to their low abundance and masking by the abundant proteins like RuBisCO. Therefore, to increase the proteome coverage, RuBisCO was removed, There are reports of successful RuBisCO removal by PEG precipitation (Xi et al., 2006), affinity purification (Cellar et al., 2008), higher DTT concentrations (Cho et al., 2008), Ca^2+^/phytate fractionation (Krishnan and Natarajan, 2009) and protamine sulfate precipitation (Kim et al., 2013). Here, PEG precipitation and RuBisCO IgY affinity chromatography were used for RuBisCO removal and S-nitrosylation analysis.

### Immunoaffinity removal of RuBisCo and MS identification of the affinity purified S-nitrosylated proteins from the RuBisCo depleted fractions

**Figure 2:**
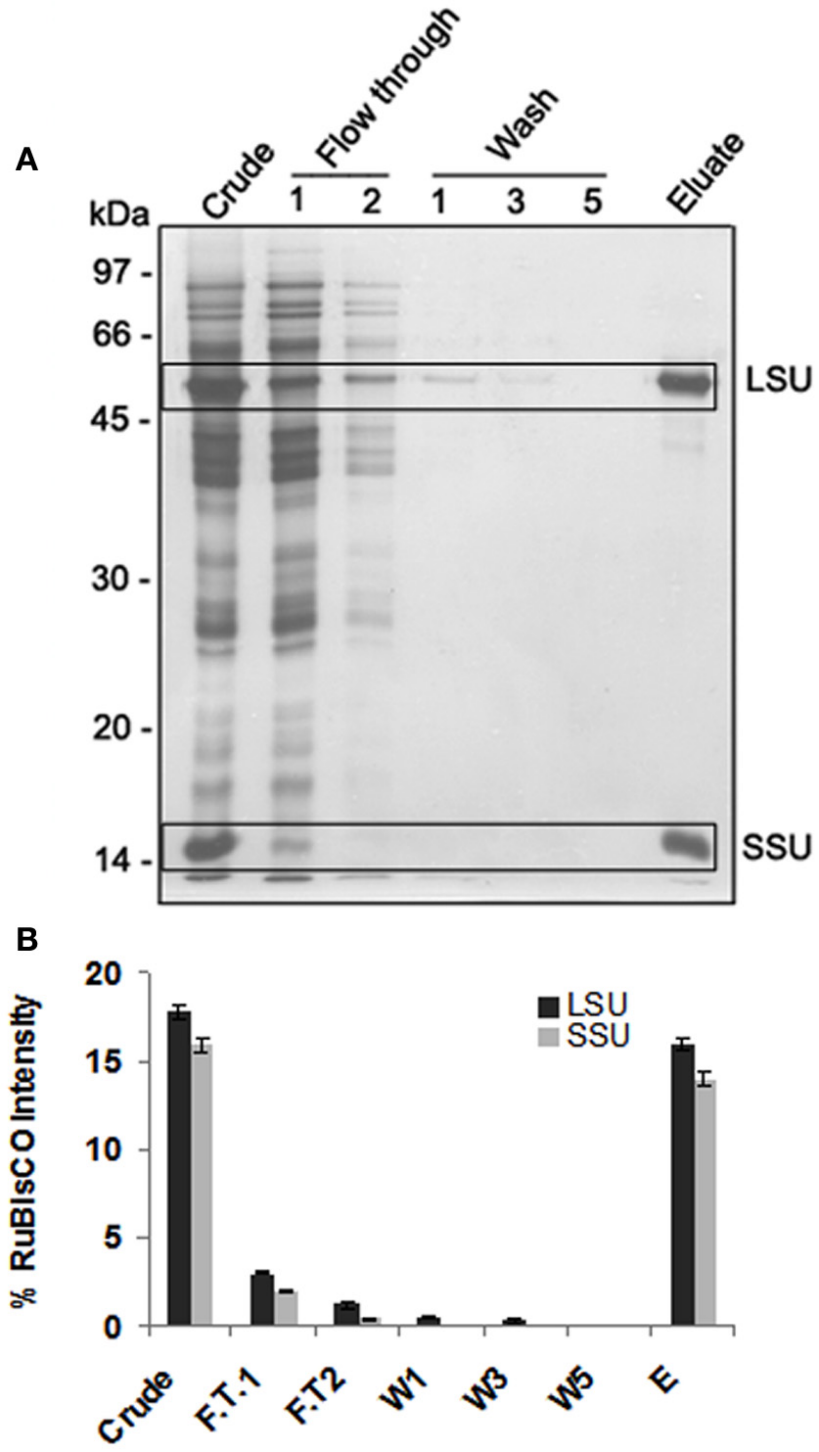
**1-DE of RuBisCO depleted fractions and its comparison with the crude proteins displaying substantial removal of RuBisCO. (A)** SDS-PAGE (12%) gel showing RuBisCO depletion after immunoaffinity purification using Seppro IgY-RuBisCO spin column kit. Large (LSU) and small (SSU) subunit of RuBisCO are marked in boxes. **(B)** Relative intensity of the polypeptides of LSU and SSU of RuBisCO quantified using densitometric scanning (AlphaImager software, Alpha Innotech Corporation). Polypeptide intensities were calculated by subtracting the background intensity. The results are representative of three biological replicates.

PEG precipitation was not effective as along with RuBisCO other proteins were also depleted (data not shown). Seppro RuBisCO spin columns (IgY affinity purification) removed 83% and 87.5% of large and small subunit of RuBisCO respectively as shown by the densitometric quantification (Figures 2A,B). For the S-nitrosylation analysis, RuBisCO depleted fraction, F.T.1 (flow through 1) was used.

**Figure 3:**
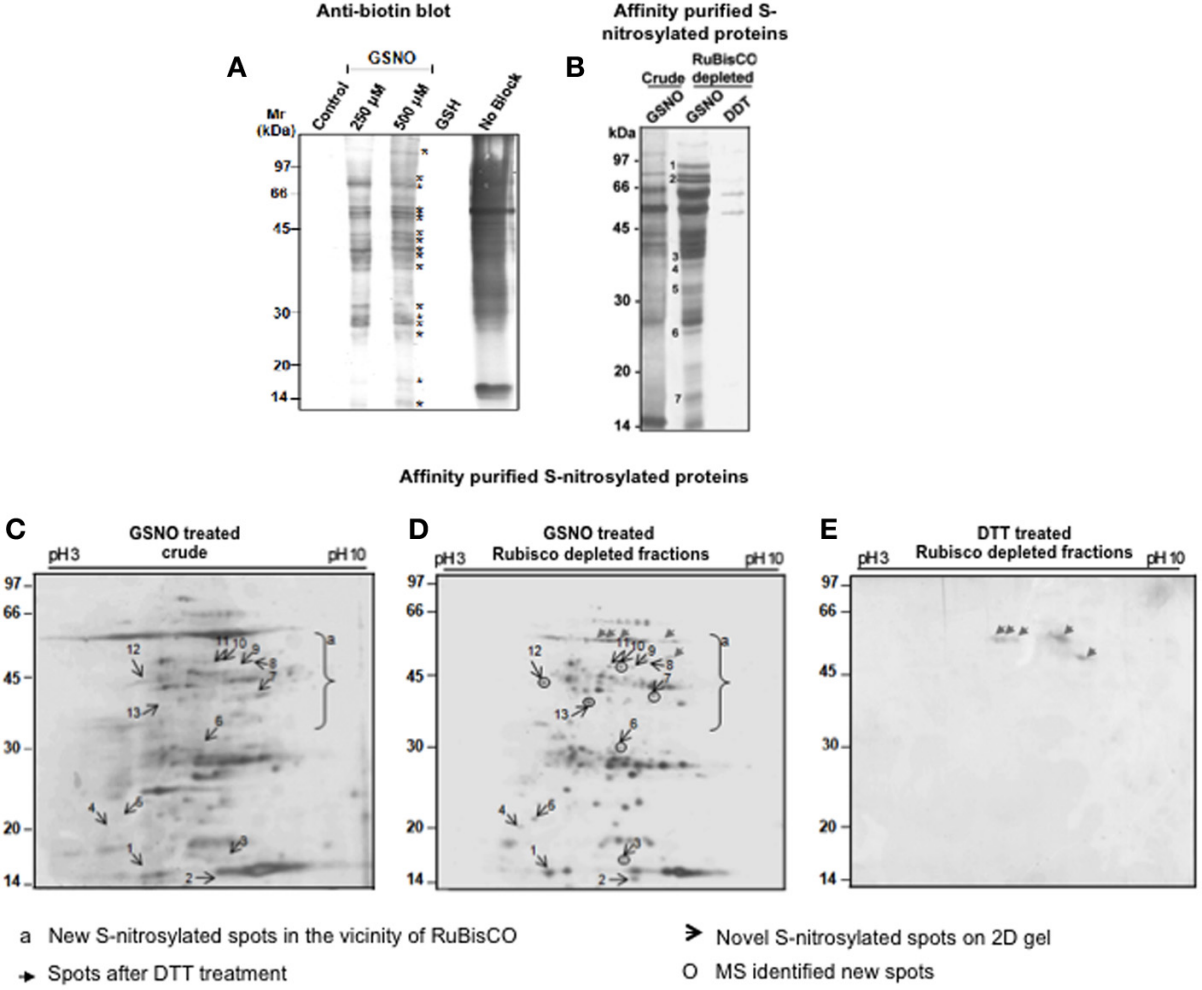
**Detection and purification of the S-nitrosylated proteins from the RuBisCO depleted fractions. (A)** RuBisCO depleted extracts containing 250 μg protein were treated with or without 250 μM and 500 μM GSNO or GSH (250 μM) and labeled with biotin using biotin switch technique. Additionally, proteins without MMTS (no block) treatment served as a control for the blocking step. Proteins were resolved on a 12% SDS-PAGE gel and blotted onto nitrocellulose membrane. Biotinylated proteins are marked with asterisk (*). **(B)** For the purification of the S-nitrosylated proteins, proteins (5 mg) treated with GSNO (500 μM) were subjected to biotin switch method, followed by their purification using neutravidin affinity chromatography. As a negative control, proteins were first S-nitrosylated with GSNO (500 μM) and then reduced with 10 mM DTT. Eluates were separated on a 12% SDS-PAGE gel. Purified S-nitrosylated polypeptides absent in the crude are marked with numbers. **(C)** and **(D)** 2-D gels (12%) of purified S-nitrosylated proteins from the crude and RuBisCO depleted fractions and after GSNO (500 μM) treatment. **(E)** 2-D gel (12%) of the GSNO (500 μM) and DTT (10 mM) treated RuBisCO depleted fractions. Gels were silver stained by MS compatible silver staining and analyzed using ImageMaster 2-D Platinum software.

S-nitrosylated proteins were detected in the RuBisCO depleted fractions by BST. The RuBisCO depleted fractions (0.8 μg/ul) were dissolved in the HEN buffer and GSNO was used for mimicking the *in vivo* S-nitrosylation. Immunoblot of GSNO (250 and 500 μM) treated fractions showed 17 immunopositive polypeptides (Figure 3A, marked with *) which were absent in the control and GSH treated fractions (250 μM, an inactive analog of GSNO), suggesting specific S-nitrosylation. Omission of the blocking (positive control), showed non-specific biotinylation of the free thiols indicating good efficacy of the procedure.

Affinity purified S-nitrosylated proteins showed 16 polypeptides on a 12% gel (Figure 3B), including 7 polypeptides (Figure 3B, marked with numericals) which were absent in the crude, showing that these were competed out by RuBisCO. The DTT-treated fraction showed only two polypeptides (52 and 60 kDa) indicating reversibility of the reaction.

**Figure 4:**
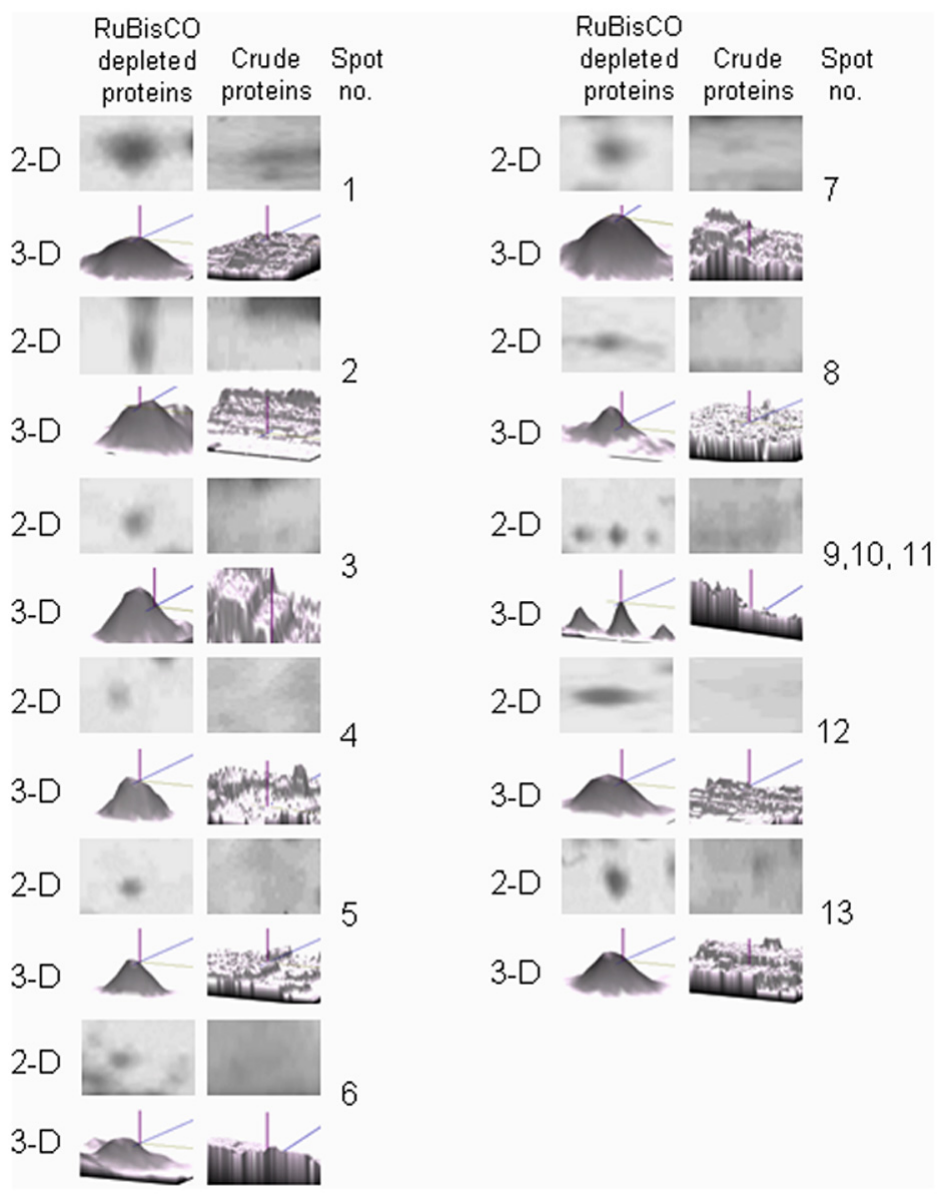
**Three-dimensional (3-D) view of unique protein spots in the RuBisCO depleted fractions generated using ImageMaster 2-D Platinum software (GE Healthcare)**.

RuBisCO depleted purified S-nitrosylated proteins resolved as 110 spots, while crude S-nitrosylated proteins showed 97 spots on the 2-D gel (Figures 3C,D). DTT treatment (a negative control) showed five spots (Figure 3E, marked with gray arrows), molecular weight of which corresponded with the DTT treated polypeptides (Figure 3B), showing repeatability of the results. A spot-to-spot comparison and statistical analysis using the ImageMaster 2-D Platinum software, detected 13 new spots in the RuBisCO depleted fractions with a significant (*p*<0.05) change in the abundance (Figures 3C,D). A three-dimensional view of these spots confirmed their increased abundance (Figure 4). Interestingly, seven (spot 7, 8, 9, 10, 11, 12, and 13), of these spots were in the vicinity of RuBisCO as seen on the 2-D gel of crude and RuBisCO depleted S-nitrosylated proteins (Figures 3C,D, marked with a), showing that RuBisCO masked these spots in the crude. These spots were identified using nLC-MS/MS as peptidyl-prolyl cis-trans isomerase (PPIase), malate dehydrogenase and fructose-bisphosphate aldolase (Table 1).

**Table 1:** **S-nitrosylated proteins identified from NO donor (GSNO) treated RuBisCO depleted fractions of *B. juncea***.

**Protein name**	**Acc. no.**	**M.S.**	**S.C.(%)**	**Matched peptides [Identified peptide (ion score)]**	**MW(Da)/pi**		**Spot number**	**Reported as S-nitrosylated in crude *B.juncea***	**Reported as S-nitrosylated in any other study**	**Functional category**
					**Observed**	**Theoretical**				
Thioredoxin H-type (*Brassica rapa*)	gi|11135129	106	8%	1[VDVDELATVAK (106)]	15000/7.3	13749/5.3	03	No	Maldonado-Alconada et al., 2010; Kato et al., 2012; Lin et al., 2012	Stress/signaling/redox
**Predicted protein, (Salt responsive protein 2, *Hordeum vulgare****)*	gi|326514010 (NP_001234 228)	51	5%	2[LATGEPLR (36)]	29870/7.0	51137/8.7	06	No	–	
Hypothetical protein ARALYDRAFT_486711 (Peptidyl-prolyl cis-trans isomerase CYP20-3, *Arabidopsis lyrata*)	gi|297817542 (NP_191762)	113	11%	3[IVMGLFGEVVPK(52)]	43200/8.3	26725/8.6	07	No	Lindermayr et al., 2005; Tanou et al., 2012	
Myrosinase (*Brassica napus*)	gi|127733	102	3%	2[GYAIGTDAPGR (79)]	51480/6.8	63266/6.6	10	Yes	–	
Malate dehydrogenase, mitochondrial precursor (*Brassica napus*)	gi|2497857	240	15%	5[SQVVGYMGDDNLAK (71)]	42510/4.7	35860/8.8	12	No	Lindermayr et al., 2005; Tanou et al., 2012	Metabolism
Fructose-bisphosphate aldolase, class I (*Arabidopsis thaliana*)	gi|18420348	457	15%	6 [LDSIGLENTEANR(95)]	38054/5.9	43029/6.7	13	Yes	Lindermayr et al., 2005; Maldonado-Alconada et al., 2010; Tanou et al., 2012	

Protein identification was performed by nLC-MS/MS using Mascot search engine using NCBI database. Spots were identified from Figure 3D. Proteins in bold indicate novel S-nitrosylation targets which are not identified in any plant system. The best matching peptide identifying the protein with ion score is reported, M.S., Mowse Score; Acc. no., Accession number; Seq. Cov., Sequence coverage.

### Cold responsive proteins from the RuBisCo depleted fractions showed stress/signaling/redox related function as a major category of the S-nitrosylated proteins

We have earlier shown that cold stress modulated S-nitrosylation and few targets were identified (Abat and Deswal, 2009). To enrich the repertoire, cold responsive S-nitrosylated proteins were purified from the RuBisCO depleted fractions of the cold treated seedlings. Cold stress of 6 h was chosen as it showed maximum NO and SNO (Abat and Deswal, 2009) production. Eleven endogenously S-nitrosylated polypeptides (24-108 kDa) were resolved on the SDS-PAGE gel (Supplementary material S1, marked with numericals). These were identified as 11 proteins with a significant score (Table 2). DTT treated cold samples showed, only three faint polypeptides (Supplementary material S1, marked with *).

**Table 2:** **Cold responsive S-nitrosylated proteins from RuBisCO depleted fractions of cold treated *B. juncea* seedlings**.

**Protein name**	**Acc. no.**	**M.S.**	**S.C.(%)**	**Matched peptides [Identified peptide (ion score)]**	**MW(Da)/pl**		**Spot/polypeptide number and patter**	**Reported as S-nitrosylated in crude**	**Reported as S-nitrosylated protein in anyother study**	**Functional category**
					**Observed**	**Theoretical**				
**Putative lactoylglutathionelyase (*Brassica rapa*)**	gi|157890952	487	36%	10[IANQELGGKITR (84)]	15657/6.2	32006/5.3	2, U	No	–	Stress/signaling/redox
Daikon cysteine protease RD21 (*Raphanus sativus*)	gi|219687002	246	16%	5[NGGIDTEEDYPYK (105)]	22055/5.5	32085/4.27	4, U	No	Maldonado-Alconada et al., 2010	
**Vacuolar calcium binding protein (*Raphanus sativus*)**	gi|9049359	120	9%	2[TEETPAVVEEEK (61)]	29003/6.6	27094/4.1	8, D	No	–	
Fe-superoxide dismutase, partial (*Arabidopsis thaliana*)	gi|166700	50	6%	2[RPDYIK (28)]	41087/6.7	25409/6.3	11, U	Yes	Lin et al., 2012; Tanou et al., 2012	
*Glutathione S-transferase (Brassica napus)*	gi|87294807	160	17%	4[SPLLLQSNPIHK(52)]	95510	24887	*3, U*	Yes	Tanou et al., 2009	
*Chaperonin 10* (*Arabidopsis thaliana*)	gi|3057150	77	8%	3[YAGTEVEFNDVK (49)]	95510	26912	*3, U*	No	Lindermayr et al., 2005	
***Epithiospecifier protein (Brassica rapa)***	gi|211905345	428	33%	16[FITKLDEEGGPEAR(74)]	53640	37890	*6, U*	No	–	
*MLP*-*like protein 328* (*Arabidopsis thaliana*)	gi|18379240	108	17%	7[GLEGHVMEQLK(47)]	40430	17616	*8, U*	Yes	Lindermayr et al., 2005	
**Soluble inorganic pyrophosphatase 1 (*Arabidopsis thaliana*)**	gi|15242465	222	11%	3[MEVATDEDFTPIK(90)]	29010/7	33644/5.7	7, D	No	–	Metabolism
Putative fructose-bisphosphate aldolase (*Arabidopsis thaliana*)	gi|14539316	176	11%	3[LDSIGLENTEANR(94)]	43041/6.9	37291/8.8	12, U	Yes	Lindermayr et al., 2005; Maldonado-Alconada et al., 2010; Tanou et al., 2012	
Fructose-bisphosphate aldolase, class I (*Arabidopsis thaliana*)	gi|18420348	713	19%	I5[LDSIGLENTEANR(116)]	52132/7	43029/6.7	14, U	Yes	Lindermayr et al., 2005	
*AT4g38970/F19H22_70* (*Fructose biphosphate aldolase 1, Arabidopsis thaliana*)	gi|16226653	334	32%	I8[LDSIGLENTEANR(47)]	53640	38858	*6, U*	Yes	Lindermayr et al., 2005	
*Glyceraldehyde-3-phosphate dehydrogenase, cytosolic* (*Sinapinis alba*)	gi|120675	599	59%	25[VPTVDVSWDLTVR(75)]	53640	37015	*6, U*	No	Lindermayr et al., 2005; Maldonado-Alconada et al., 2010	
Sedoheptulose-bisphosphatase *(Arabidopsislyrata)*	gi|297816906	675	27%	18[LTGVTGGDQVAAAMGIYGPR (134)]	68081/6.2	42861/6.0	15, U	Yes	Tanou et al., 2009	Photosynthesis
*Sedoheptulose-bisphosphatase (Arabidopsislyrata)*	gi|297816906	358	22%	11[GIFTNVTSPTAK(70)]	42250	42861	*7, U*	Yes	Tanou et al., 2009	
*Beta-carbonic anhydrase, chloroplastic (Brassica napus)*	gi|297787439	374	29%	11[VISELGDSAFEDQ CGR(82)]	95510	36127	*3, U*	No	Lindermayr et al., 2005; Tanou et al., 2012	
*Oxygen-evolving enhancer protein 2, chloroplastic* (*Pisum sativum*)	gi|131390	169	10%	7[FVEDTASSFSVA(76)]	37500	28201	*9, U*	No	Lindermayr et al., 2005	
*Hypothetical protein SORBIDRAFT_02g002690, (23 kDa polypeptide of PS II*)	gi|242047384 (AAB82135)	122	8%	3[HQLITATVSDGK(63)]	37500	27718	*9, U*	No	Tanou et al., 2012	
**Unknown protein 18 *(Vitis rotundifolia)***	gi|205830697	149	100%	3[TNAENEFVTIKK((78)]	29950/5.3	1393/5.85	9. D	No	–	Unknown
**Unknown protein 18 *(Vitis rotundifolia)***	gi|205830697	158	100%	1[TNAENEFVTIK(80)]	22076/5.8	1393/5.85	5, D	No	–	
***Unnamed protein product (Thellungiella halophila)***	gi|312282755	500	53	2[(VPTVDVSWDLTVR(75)]	53640	32088	*6,U*	No	–	

**Figure 5:**
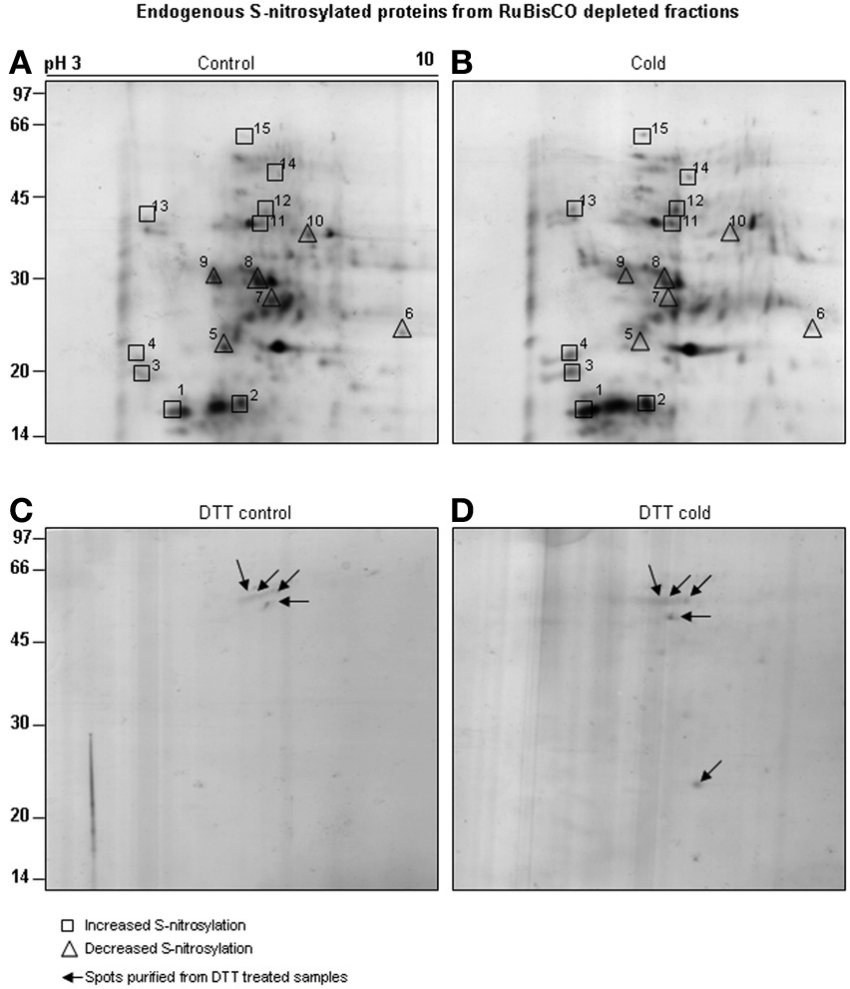
**Purification of cold responsive S-nitrosylated proteins.** RuBisCO depleted proteins (5 mg) from control **(A)** and cold **(B)** treated seedlings were subjected to BST and neutravidin affinity purification. Purified S-nitrosylated proteins were resolved on 2-D gel using non-linear IPG strips (13 cm, pH 3-10) and 12% SDS-PAGE. As a control, RuBisCO depleted proteins from the control **(C)** and cold **(D)** seedlings were treated with DTT (10 mM) and the purified spots were resolved on 12% SDS-PAGE (marked with arrows). Gels were stained using MS compatible silver staining and analyzed using ImageMaster 2-D Platinum software. Spots showing increased S-nitrosylation intensity after cold stress are marked with boxes and decreased S-nitrosylation by triangle.

*Protein identification was performed by nLC-MS/MS using Mascot search engine using NCBI database. Spots/polypeptides were identified from Figure 5B and Supplementary material S1. Proteins in bold indicate novel S-nitrosylation targets which are not identified in any plant system. Proteins in italics indicate the protein identified from 1-D gel. The best matching peptide identifying the protein with ion score is reported, M.S., Mowse Score; Acc. no., Accession number; Seq. Cov., Sequence coverage*.

Neutravidin-affinity purified cold responsive S-nitrosylated proteins resolved as 78 spots, out of which 15 spots showed differential S-nitrosylation (Figures 5A,B). Of these, 9 spots showed increased (Figures 5A,B, marked with square), while 6 spots showed decreased (Figures 5A,B, marked with triangle) S-nitrosylation. DTT treated sample showed four (Figure 5C) and five (Figure 5D) spots in the RuBisCO depleted fractions from control and cold treated seedlings. The 2-D gel showed better resolution of the low molecular weight S-nitrosylated proteins than the 1-D gels. Most abundant spots (10) showing differential S-nitrosylation (Figure 5B) were identified by nLC-MS/MS (Table 2). The difference in the theoretical and the experimental molecular weights of some of the identified proteins could be due to different isoforms, PTMs or degradation of the proteins.

Overall, the functional categorization of the cold responsive S-nitrosylated targets showed stress/signaling/redox related functions to be the largest functional category. The second largest category was of metabolic proteins. The third category included photosynthetic targets, while unknown targets were least in number. It is worth mentioning that putative lactoylglutathione lyase/glyoxylase I (Gly I), epithiospecifier protein, vacuolar calcium binding protein, inorganic pyrophosphatase I, unnamed protein products and unknown proteins are identified as S-nitrosylated proteins for the first time in plants.

**Figure 6:**
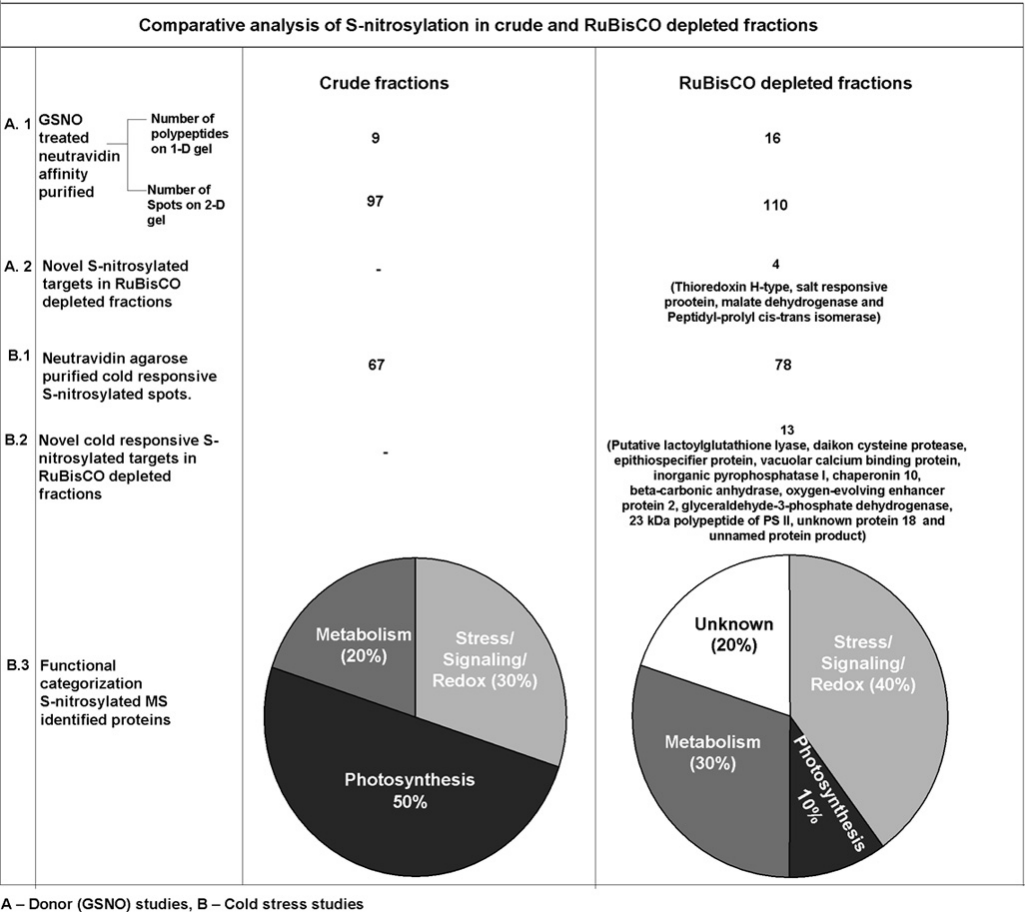
**Comparison of S-nitrosylation in crude and RuBisCO depleted fractions showing increased S-nitrosoproteome coverage by RuBisCO depletion**.

A comparison of S-nitrosylation of the crude with RuBisCO depleted fractions showed that RuBisCO depletion increased polypeptide/spot number on the 1-D/2-D gels, indicating its effectiveness in S-nitrosylation analysis (Figure 6). MS identification further supported the results, as novel targets were identified in the GSNO and cold treated RuBisCO depleted fractions. Moreover, the functional categorization of the cold responsive S-nitrosylated proteins showed a shift from the photosynthetic targets to the redox/stress/signaling and metabolic proteins in the RuBisCO depleted fractions in comparison with the crude. This suggests a functional switching over from the normal physiology to signaling for combating the stress. Interestingly, a new category of unknown proteins was also observed in RuBisCO depleted fractions. Therefore, RuBisCO depletion seems to be a fruitful strategy in unraveling the physiological functions of S-nitrosylation and in enhancing the S-nitrosoproteome coverage.

### Effect of nitric oxide and cold stress on superoxide dismutase activity

**Figure 7:**
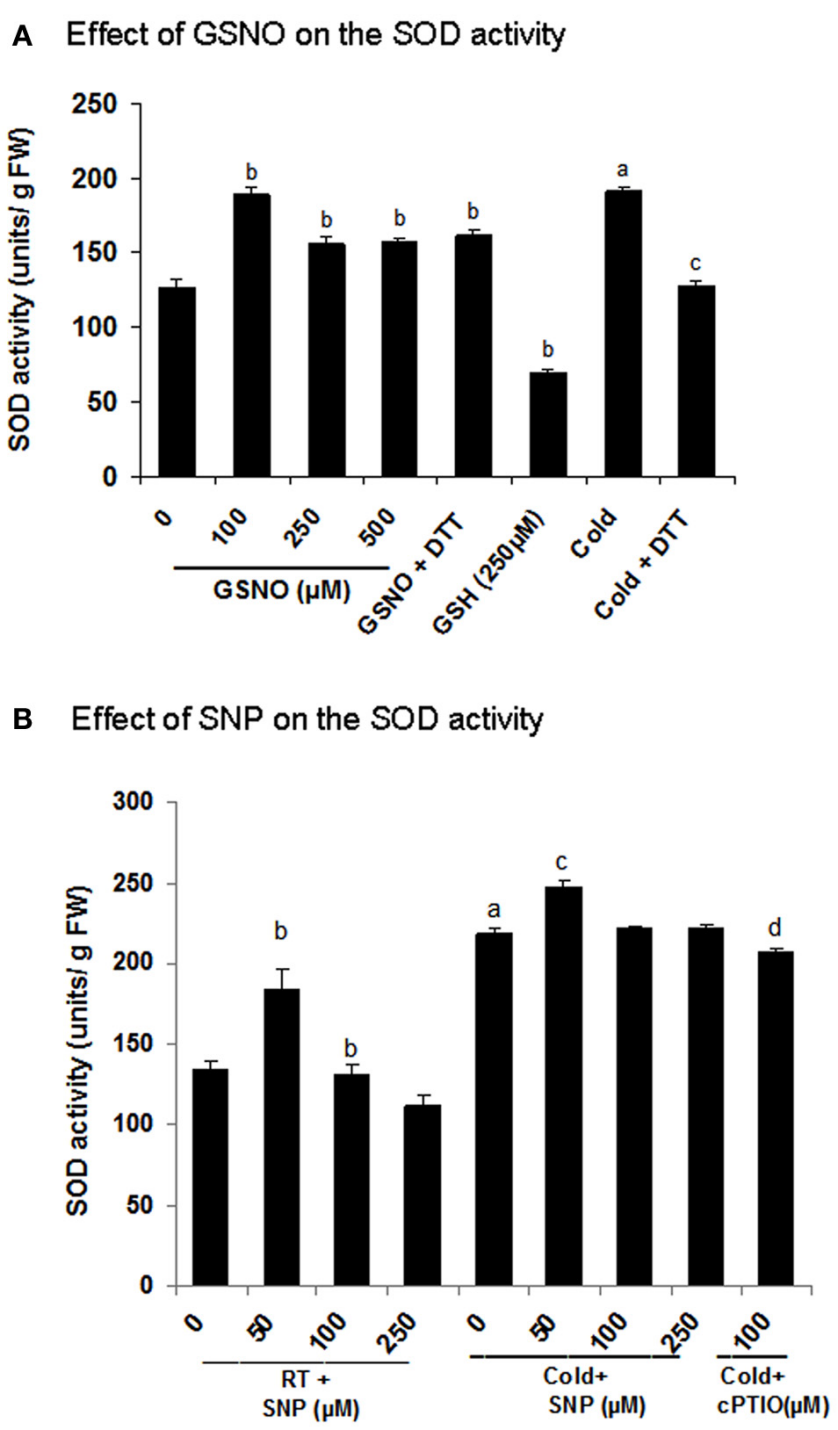
**Functional validation of superoxide dimutase (SOD) as a cold responsive S-nitrosylated protein. (A,B)** Effect of GSNO and SNP on the SOD activity measured using NBT reduction assay. For the *in vitro* assays, extracts were incubated with or without GSNO (100 μM, 250 μM and 500 μM) or GSH (250 μM) prior to the activity analysis. Incubation with DTT (10 mM) was also done after GSNO (100 μM) treatment to check the reversal. Seedlings were treated with SNP (50–250 μM) and cPTIO (100 μM) with or without cold stress. Extracts from these samples were used for the assay. Error bars represents standard deviation from three independent experiments (biological repeats) performed in triplicates (technical repeats). Statistical significance was determined by Student's *t*-test. In **(A)** statistically significant difference (*p* < 0.05) between RT (control) and cold is shown (by a), control and GSNO (by b) and cold and DTT (by c). In **(B)** values with the same alphabets are showing significant difference (*p* < 0.05) between RT and cold (by a), control and SNP (by b), cold and SNP (by c) and cold and cPTIO (by d).

In the present study, Fe-SOD showed an increase in S-nitrosylation in cold (spot number 11, Figures 5A,B and Table 2). To know, the effect of S-nitrosylation on the SOD activity, the extracts were incubated with GSNO (100 μM), which showed 49% increase in the activity (Figure 7A). Cold treatment showed 50% increase in the SOD activity. DTT (10 mM) brought down the activity to 27 and 33% in the GSNO and cold treated samples respectively. As DTT treatment did not show 100% reversal, this indicated the role of other NO based PTMs, besides S-nitrosylation in regulating SOD. To further confirm these results, NO donor (SNP) and inhibitor (cPTIO) treatment was given to control (RT) and cold (4°C) treated seedlings and the extract was used for the activity assay. SNP (50 μM) increased the activity to 84.1% in cold, while it was not promoting the activity at 100 and 250 μM (Figure 7B). Control showed a similar trend. cPTIO reduced the increased activity to the basal level. These results showed cold stress mediated SOD activation by S-nitrosylation.

## Discussion

Recently, the role of NO as a key component in cold stress signaling was emphasized (Liu et al., 2010; Gupta et al., 2011; Bai et al., 2012; Wang et al., 2012; Sehrawat et al., 2013). In the present report, evidences for the NO signaling in cold stress are provided. Endogenous NO increased by 2 fold after 6 h of cold stress and NR seems to be a major contributor in the NO production. NR dependent NO production in cold was earlier shown in *A. thaliana l*eaves (Cantrel et al., 2011) and *Baccaurea ramiflora* seeds (Bai et al., 2012). NOS-like enzyme dependent NO production in cold stress is reported in *Pisum sativum* leaves (Corpas et al., 2008), *Chorispora bungeana* suspension cultures (Liu et al., 2010), *Solanum lycopersicum* fruits (Zhao et al., 2011), *B. juncea* seedlings (Talwar et al., 2012) and *Camellia sinensis* pollen tubes (Wang et al., 2012). The enzymes involved in cold induced NO production vary with plant system, tissue type and stress, indicating differential regulatory mechanism(s) of NO production.

Cold stress alters the cellular redox homeostasis, while thiols play a significant role in its maintenance. In this study, an increase in the available thiol groups in cold was observed, indicating a shift from the buried to the available thiols, probably due to conformational change in the proteins. Interestingly, protein based thiols constituted 57% of the cold modulated total thiols, while low molecular weight thiols constituted 43%, indicating that both are contributing almost equally in maintaining the redox homeostasis. Unlike protein based thiols, low molecular weight thiols showed a decrease after cold as observed in heat treated pea seedlings (Ivanov and Kerchev, 2007) and cadmium treated *Salsola kali* leaves (Rosa et al., 2005). This decrease could be due to the utilization of low molecular weight thiols in S-nitrosylation of proteins. To establish this, S-nitrosylation was analyzed.

The major hurdle in the S-nitrosylation analysis in cold stress treated seedlings was RuBisCO, the most abundant S-nitrosylated protein. It competes with other S-nitrosylated proteins and hinders their resolution and MS identification. Therefore, RuBisCO (more than 80%) was removed from *B. juncea* crude extracts using immunoaffinity purification as it is quite conserved across the plant species.

To test, if RuBisCO removal improves efficacy of S-nitrosylation analysis, BST of RuBisCO depleted fractions (using GSNO) was performed. It improved the protein resolution as 7 new polypeptides (on the 1-D gel) and 13 new spots (on the 2-D gel) were observed. Increased polypeptide/spot number also suggests improved efficacy of the BST and neutravidin affinity chromatography. Moreover, it also enhanced the identification of the regulatory targets (thioredoxin, salt-responsive protein, PPIases and malate dehydrogenase), which earlier escaped detection in the crude (Abat and Deswal, 2009).

Cold stress increased the S-nitrosylation of Gly I, cysteine protease, Fe-SOD and fructose biphosphate aldolase, while decreased the S-nitrosylation of vacuolar calcium binding proteins, inorganic pyrophosphatase and unknown proteins (Table 2). Overall, the S-nitrosoproteome coverage of cold stress responsive signaling and redox related targets was increased by RuBisCO depletion.

In the present study, it is shown that cold induced NO causes increased S-nitrosylation of SOD and contributes to superoxide dismutation and ROS detoxification. S-nitrosylation of Fe-SOD was also shown in the salinity treated citrus leaves (Tanou et al., 2012), while Cu/Zn SOD was identified as a S-nitrosylated target in Arabidopsis (Lindermayr et al., 2005) and rice (Lin et al., 2012). This data is consistent with the previous report where increased S-nitrosylation of the enzymes of ascorbate glutathione cycle [ascorbate peroxidase, glutathione reductase and dehydroascorbate reductase (DHAR)] reduced desiccation-induced ROS accumulation and eventually enhanced the desiccation tolerance in *Antiaris toxicaria* seeds (Bai et al., 2011). Besides SOD, thioredoxin (H-type) is also S-nitrosylated and acts as a redox regulator of the transcription factors including non-expressor of pathogenesis related protein (NPR1, Tada et al., 2008), which further regulates the expression of the defense responsive genes. Overexpression of thioredoxin (H-type) in transgenic rice, induced the expression of chaperones in seeds (Wakasaa et al., 2013).

The identified proteins also include novel S-nitrosylated targets like Gly I, a vacuolar calcium binding protein (CaB) and inorganic pyrophosphatase 1. Although, tyrosine nitration of Gly I in salt stress was shown in citrus (Tanou et al., 2012), this is the first report of S-nitrosylation of Gly I in plants. The identified CaB (involved in maintaining calcium homeostasis) showed similarity with a unique CaB from *Raphnus sativus* (Yuasa and Maeshima, 2000). S-nitrosylation of CaB, suggest a cross-talk between NO and calcium signaling. To the best of our knowledge, till date the role of this unique CaB is not investigated in stress, therefore it would be interesting to analyze its role in calcium signaling in cold. Overexpression of Arabidopsis inorganic pyrophosphatase in *E. coli*, conferred enhanced tolerance to abiotic stress (Yoon et al., 2013).

Identification of myrosinase and epithiospecifier protein (a novel S-nitrosylated protein), involved in the glucosinolates hydrolysis as targets, suggest the role of S-nitrosylation in regulating “glucosinolate hydrolysis pathway.” This pathway is specific to Brassicaceae and is involved in protection against abiotic stress (Martinez-Ballesta et al., 2013).

Three enzymes of Calvin cycle namely fructose biphosphate aldolase, sedoheptulose-1,7-bisphosphatase and GAPDH were identified as cold responsive S-nitrosylated targets. Fructose biphosphate aldolase, is a cold responsive protein (Hashimoto and Komatsu, 2007). In the present study, an increase in the S-nitrosylation in cold was observed (spot number 12 and 14, Figures 5A,B and Table 2). Fructose biphosphate aldolase activity with GSNO (a NO donor), showed a dose dependent increase, while treatment with GSH (an inactive analog of GSNO) had no effect (Supplementary material S2). DTT (a reductant) reduced the activity back to the control level. These results showed a positive regulation of fructose biphosphate aldolase by S-nitrosylation. Increased activity of sedoheptulose-1,7-bisphosphatase enhanced salt stress tolerance in transgenic rice seedlings (Feng et al., 2007). Re-localization of GAPC1 (an isoform of cytosolic GAPDH) to the nucleus in cadmium treated *A. thaliana* seedlings, indicated its role in stress signaling (Vescovi et al., 2013).

Interestingly, after the RuBisCO removal six hypothetical/uncharacterized/unnamed proteins were identified. These were searched in NCBInr protein database using BLASTP. Hypothetical protein ARALYDRAFT_486711, hypothetical protein SORBIDRAFT_02g002690 and predicted protein were identified as PPIase, a 23 kDa polypeptide of PS II and a salt responsive protein 2 respectively. However, unknown protein 18 and unnamed protein product could not be identified, probably these are not yet reported. PPIases identified in this study are ubiquitous proteins, mediating protein folding in cold stress (Budiman et al., 2011). Additionally, ROC4 (only cyclophilin in the stroma of the chloroplast) is shown to have PPIases activity and is involved in the repair of photo-damaged PSII in *A. thaliana* (Cai et al., 2008). Chaperonin besides modulating protein folding, also regulates Fe-SOD activity (Kuo et al., 2013).

Most of the validated targets for S-nitrosylation are negatively regulated by S-nitrosylation [as reviewed by Astier et al. (2012)]. In contrast, there are very few targets like TGA1 (Lindermayr et al., 2010), ascorbate peroxidase (Bai et al., 2011), glutathione reductase (Bai et al., 2011) and DHAR (Bai et al., 2011) which are positively regulated by S-nitrosylation.

The novel targets were searched to detect other redox based PTMs using RedoxDB (http://biocomputer.bio.cuhk.edu.hk/RedoxDB/index.php), a database of protein oxidative modifications. No other redox modification was identified supporting that these targets are not yet reported and are novel in plants.

**Figure 8:**
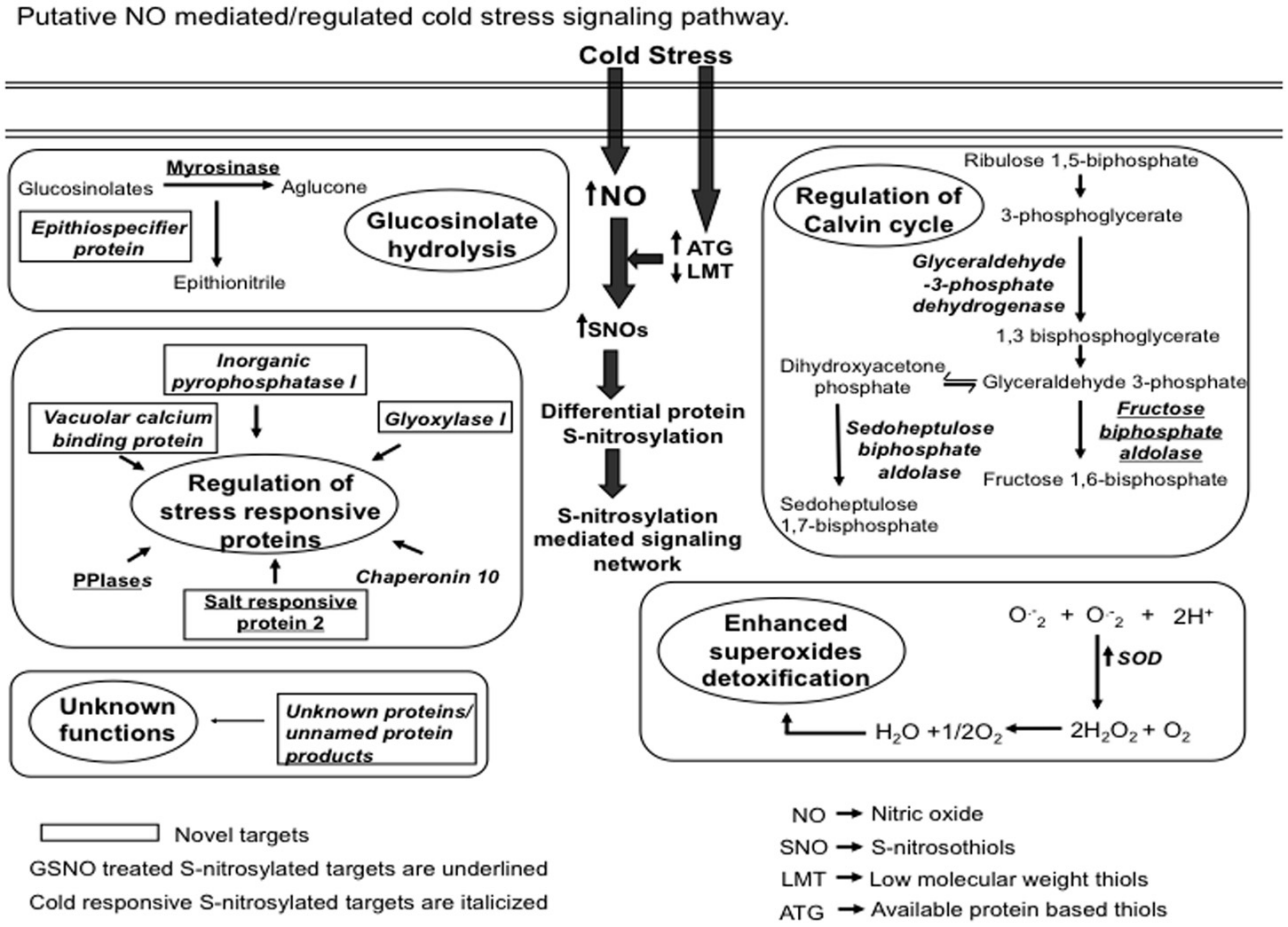
**A proposed model showing the S-nitrosylation mediated cold stress signaling.** Cold stress increased nitric oxide (NO) production. This increased NO reacts with low molecular weight thiols (LMT) such as glutathione to produce S-nitrosoglutathione (GSNO). Available thiol groups (ATGs) also showed an increase in response to the cold stress. GSNO reacts with these ATGs to produce S-nitrosothiols (SNOs). Increased SNOs promote the S-nitrosylation of constitutive as well as the regulatory proteins. S-nitrosylation of superoxide dismutase (SOD) reduces the cellular damage caused by reactive oxygen species by scavenging superoxide radicals (O^−^_2_). S-nitrosylation of myrosinase and epithiospecifier protein suggests the probable role of NO in regulating glucosinolates hydrolysis pathway. Identification of vacuolar calcium binding protein, glyoxylase I, peptidyl-prolyl cis-trans isomerase (PPIases) and chaperonin 10 could be associated with the regulation of stress responses. The proposed model also reflects the physiological relevance of S-nitrosylation in regulating the Calvin cycle.

To conclude, an increase in the NO production in cold suggested its role in maintaining cellular redox homeostasis in *B. juncea*. Cold induced NO reacts with low molecular weight thiols and promotes SNOs formation leading to S-nitrosylation. The fact that 17 new S-nitrosylated targets (4 GSNO treated and 13 cold responsive) were identified, which were not detected in crude (Abat and Deswal, 2009) suggest that these targets were more accessible for the purification and MS identification after RuBisCO depletion. The identified targets belong to multiple plant responses including redox homeostasis, glucosinolate hydrolysis pathway, stress signaling and Calvin cycle as described in Figure 8. Thus, indicated the role of accumulated NO in orchestrating these cellular responses through S-nitrosylation. Therefore, RuBisCO depletion is suitable for downstream proteomic analysis and could be used for the detection of other PTMs of cold responsive proteins that possibly are difficult to detect due to the abundance and fragmentation of RuBisCO in cold.

## Conflict of interest statement

The authors declare that the research was conducted in the absence of any commercial or financial relationships that could be construed as a potential conflict of interest.
