# Expression of Concern: RuBisCO depletion improved proteome coverage of cold responsive S-nitrosylated targets in *Brassica juncea*

**DOI:** 10.3389/fpls.2026.2017278

**Published:** 2026-10-14

**Authors:** Frontiers Editorial Office

With this notice, Frontiers states its awareness of concerns regarding the image integrity of **Figure 4** in “RuBisCO depletion improved proteome coverage of cold responsive S-nitrosylated targets in *Brassica juncea*”, published on 02 September 2013.

Frontiers conducted a post-publication investigation in accordance with our policies and the guidelines of the Committee on Publication Ethics (COPE). The authors were contacted and provided a response; however, due to the elapsed time, the original raw data and ImageMaster software outputs are no longer available. An institutional review has been requested from authors’ institution. The situation will be updated if more information comes to light. Frontiers would like to thank Dr Elizabeth Bik for contacting the journal regarding the published article.

