# Supplementary material for: RuBisCO depletion improved proteome coverage of cold responsive S-nitrosylated targets in Brassica juncea
Source: Front Plant Sci. 2013 Sep 2;4:342. doi: 10.3389/fpls.2013.00342 (PMC3759006; doi:10.3389/fpls.2013.00342)
Supplement: Supplementary file 1 [file DataSheet1.PDF]

Mascot Search Results

Peptide View

Spot no. 3, FIGURE 3D

MS/MS Fragmentation of **VDVDELATVAK**
Found in **gi|11135129** in **NCBInr**, RecName: Full=Thioredoxin H-type; Short=Trx-H

Match to Query 90: 1159.545448 from(580.780000,2+) intensity(4787326.0000) index(8)
Title: Cmpd 9, +MSn(580.6), 28.3 min
Data file DU-JAS-FT-1.mgf

Click mouse within plot area to zoom in by factor of two about that point

Or, Plot from 150 to 1100 Da Full range

Label all possible matches Label matches used for scoring

Show Y-axis

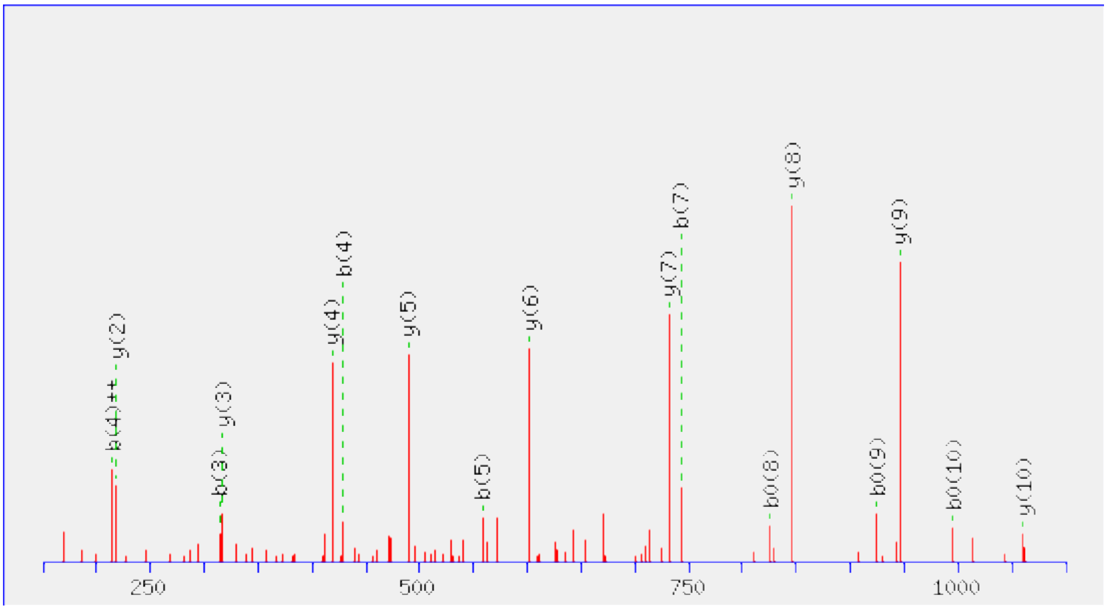

Monoisotopic mass of neutral peptide Mr(calc): 1158.6132
Fixed modifications: Carbamidomethyl (C) (apply to specified residues or termini only)
Ions Score: 106 **Expect:** 1.3e-07
Matches : 18/92 fragment ions using 18 most intense peaks

Mascot Search Results

Peptide View

Spot no. 6, FIGURE 3D

MS/MS Fragmentation of **LATGEPLR**
Found in **gi|326514010** in **NCBInr**, predicted protein [Hordeum vulgare subsp. vulgare]

Match to Query 33: 855.505448 from(428.760000,2+) intensity(4294990.0000) index(5)
Title: Cmpd 6, +MSn(429.0), 23.3 min
Data file DU-JAS-FT4.mgf

Click mouse within plot area to zoom in by factor of two about that point

Or, Plot from 150 to 750 Da Full range

Label all possible matches Label matches used for scoring

Show Y-axis

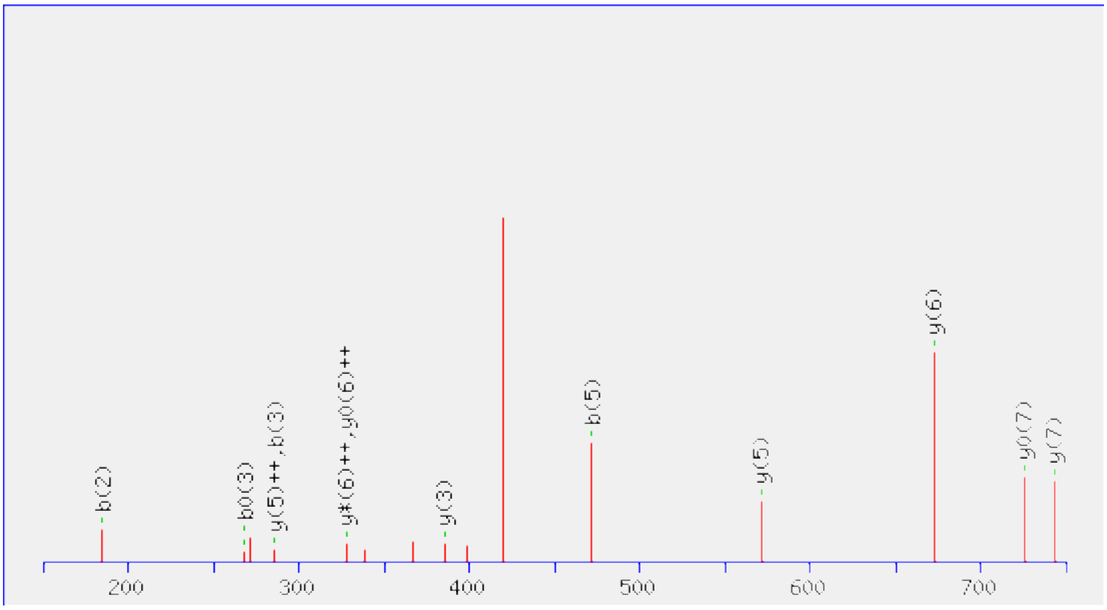

Monoisotopic mass of neutral peptide Mr(calc): 855.4814
Fixed modifications: Carbamidomethyl (C) (apply to specified residues or termini only)
Ions Score: 36 **Expect:** 1.6
Matches : 12/60 fragment ions using 13 most intense peaks (help)

Mascot Search Results

Peptide View

Spot no. 7, FIGURE 3D

MS/MS Fragmentation of **IVMGLFGEVVPK**
Found in **gi|297817542** in **NCBInr**, hypothetical protein ARALYDRAFT_486711 [Arabidopsis lyrata subsp. lyrata]

Match to Query 161: 1304.865448 from(653.440000,2+) intensity(959316.0000) index(76)
Title: Cmpd 77, +MSn(654.6), 36.7 min
Data file DU-JAS-FT3.mgf

Click mouse within plot area to zoom in by factor of two about that point
Or, Plot from 200 to 1500 Da Full range
Label all possible matches Label matches used for scoring
Show Y-axis

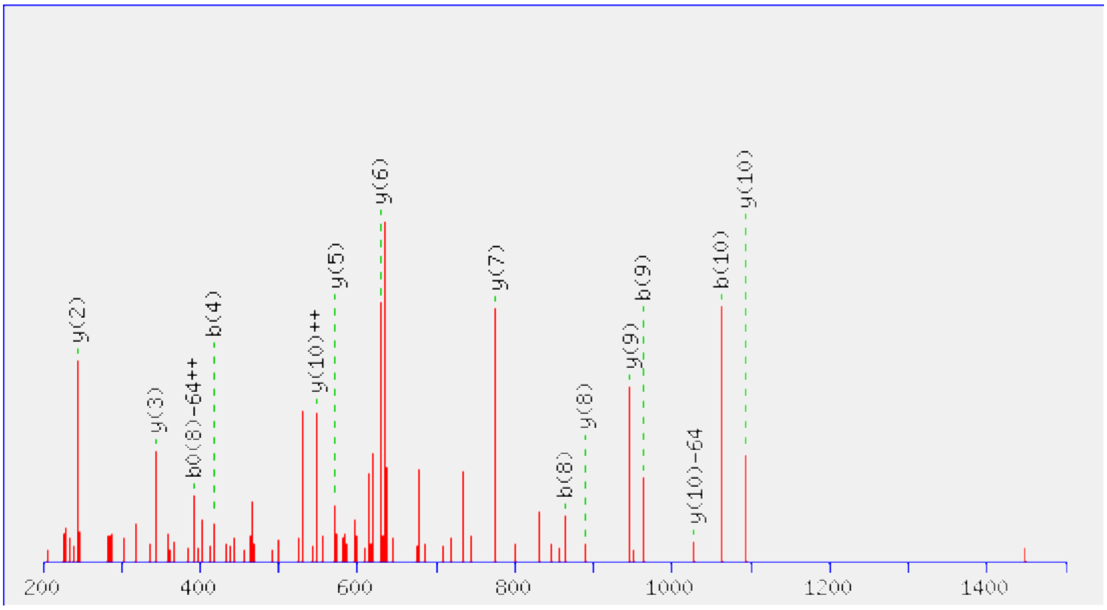

Monoisotopic mass of neutral peptide Mr(calc): 1303.7210
Fixed modifications: Carbamidomethyl (C) (apply to specified residues or termini only)
Variable modifications:
M3 : Oxidation (M), with neutral losses 0.0000(shown in table), 63.9983
Ions Score: 52 **Expect:** 0.03
Matches : 15/126 fragment ions using 27 most intense peaks

Mascot Search Results

Peptide View **Spot no. 10, FIGURE 3D**

MS/MS Fragmentation of **GYAIGTDAPGR**
Found in **gi|127733** in **NCBInr**, RecName: Full=Myrosinase; AltName: Full=Sinigrinase; AltName: Full=Thioglucosidase; Flags: Precursor

Match to Query 57: 1076.885448 from(539.450000,2+) intensity(2724479.0000) index(1)
Title: Cmpd 2, +MSn(539.4), 20.7 min
Data file DU-JAS-FT-7.mgf

Click mouse within plot area to zoom in by factor of two about that point

Or, Plot from 150 to 900 Da Full range

Label all possible matches Label matches used for scoring

Show Y-axis

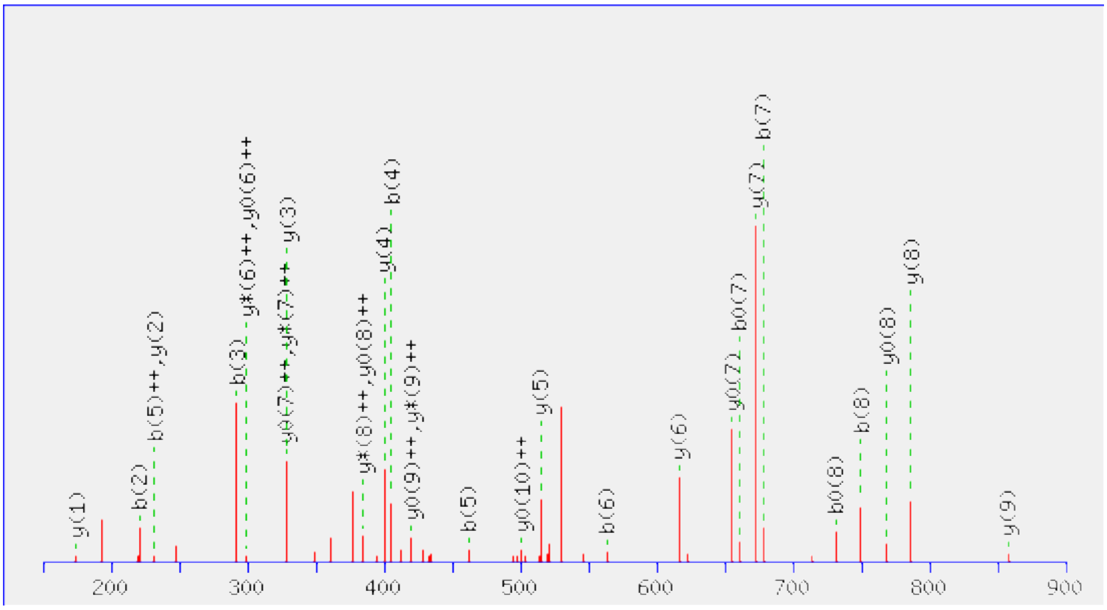

Monoisotopic mass of neutral peptide Mr(calc): 1076.5251
Fixed modifications: Carbamidomethyl (C) (apply to specified residues or termini only)
Ions Score: 79 **Expect:** 6.9e-05
Matches : 30/82 fragment ions using 36 most intense peaks (help)

Mascot Search Results

Peptide View **Spot no. 12, FIGURE 3D**

MS/MS Fragmentation of **SQVVGYMGDDNLAK**
Found in **gi|2497857** in **NCBInr**, RecName: Full=Malate dehydrogenase, mitochondrial; Flags: Precursor

Match to Query 135: 1495.865448 from(748.940000,2+) intensity(1751198.0000) index(7)
Title: Cmpd 8, +MSn(749.2), 24.5 min
Data file DU-JAS-FT-6.mgf

Click mouse within plot area to zoom in by factor of two about that point

Or, Plot from 200 to 1400 Da Full range

Label all possible matches Label matches used for scoring

Show Y-axis

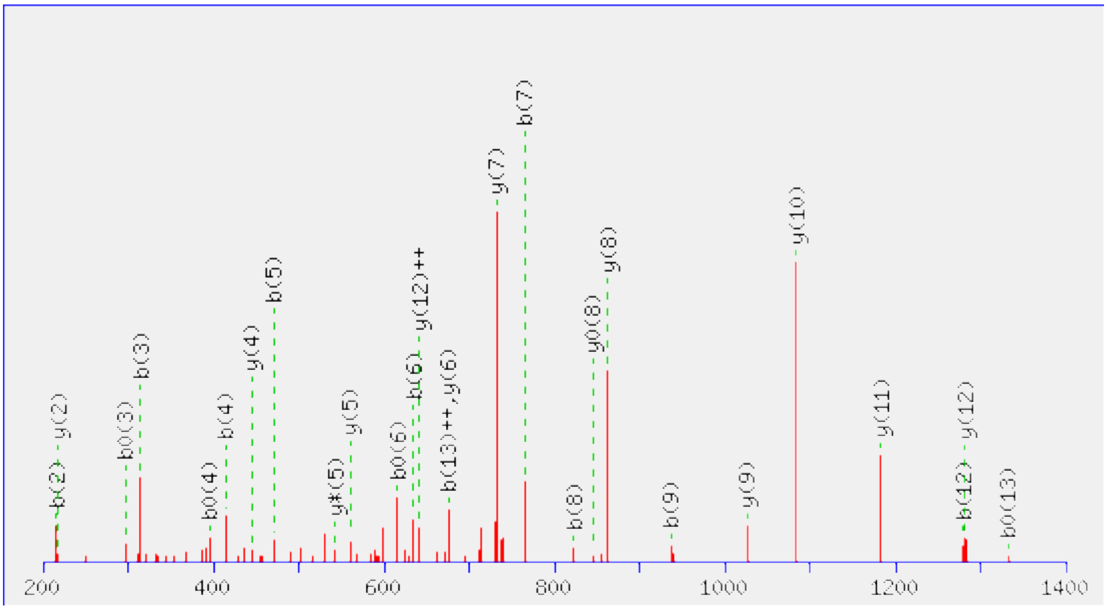

Monoisotopic mass of neutral peptide Mr(calc): 1495.6977
Fixed modifications: Carbamidomethyl (C) (apply to specified residues or termini only)
Ions Score: 71 **Expect:** 0.00042
Matches : 27/146 fragment ions using 44 most intense peaks (help)

Mascot Search Results

Peptide View

Spot no. 13, FIGURE 3D

MS/MS Fragmentation of **LDSIGLENTEANR**
Found in **gi|18420348** in **NCBInr**, fructose-bisphosphate aldolase, class I [Arabidopsis thaliana]

Match to Query 110: 1430.716090 from(716.365321,2+) index(109)
Title: File: 130406 3026E.wiff, Sample: 130406 3026E (sample number 1), Elution: 17.254 min, Period: 1, Cycle(s): 3259 (Experiment 2)
Data file DU-JAS-FT2.mgf

Click mouse within plot area to zoom in by factor of two about that point
Or, Plot from 100 to 1400 Da Full range
Label all possible matches Label matches used for scoring
Show Y-axis

Error: try setting browser cache to automatic.

Monoisotopic mass of neutral peptide Mr(calc): 1430.7001
Fixed modifications: Carbamidomethyl (C) (apply to specified residues or termini only)
Ions Score: 95 **Expect:** 2.4e-06
Matches : 19/122 fragment ions using 26 most intense peaks (help)

MATRIX SCIENCE **Mascot Search Results**

Peptide View

Spot no. 2, FIGURE 5B

MS/MS Fragmentation of **IANQELGGKITR**
Found in **gi|157890952** in **NCBInr**, putative lactoylglutathione lyase [Brassica rapa]

Match to Query 185: 1298.759960 from(650.387256,2+) index(184)
Title: File: 130406 3026I.wiff, Sample: 130406 3026I (sample number 1), Elution: 14.903 min, Period: 1, Cycle(s): 2657 (Experiment 2)
Data file F2079099.mgf

Click mouse within plot area to zoom in by factor of two about that point

Or, Plot from 100 to 1200 Da Full range

Label all possible matches ○ Label matches used for scoring ◉

Show Y-axis ☐

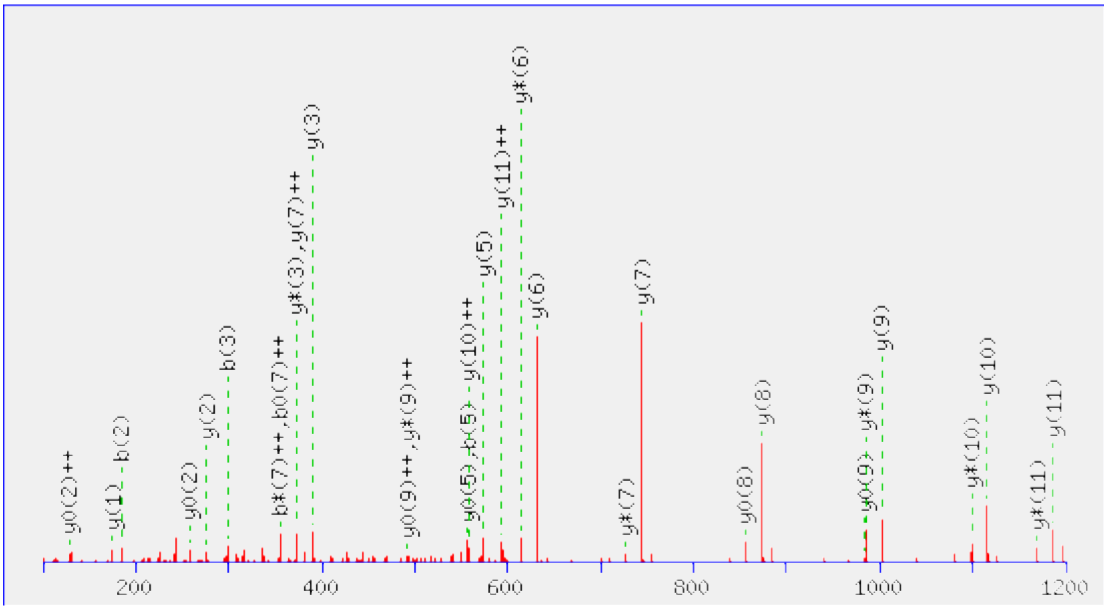

Monoisotopic mass of neutral peptide Mr(calc): 1298.7306
Fixed modifications: Carbamidomethyl (C) (apply to specified residues or termini only)
Ions Score: 84 **Expect:** 7.9e-06
Matches : 31/118 fragment ions using 44 most intense peaks (help)

MATRIX SCIENCE Mascot Search Results

Peptide View **Spot no. 4, FIGURE 5B**

MS/MS Fragmentation of **NGGIDTEEDYPYK**
Found in **gi|219687002** in **NCBInr**, daikon cysteine protease RD21 [Raphanus sativus]

Match to Query 183: 1499.682780 from(750.848666,2+) index(182)
Title: File: 130406 3026G.wiff, Sample: 130406 3026G (sample number 1), Elution: 16.037 min, Period: 1, Cycle(s): 2850 (Experiment 2)
Data file F207900.mgf

Click mouse within plot area to zoom in by factor of two about that point
Or, Plot from 100 to 1400 Da Full range
Label all possible matches Label matches used for scoring
Show Y-axis

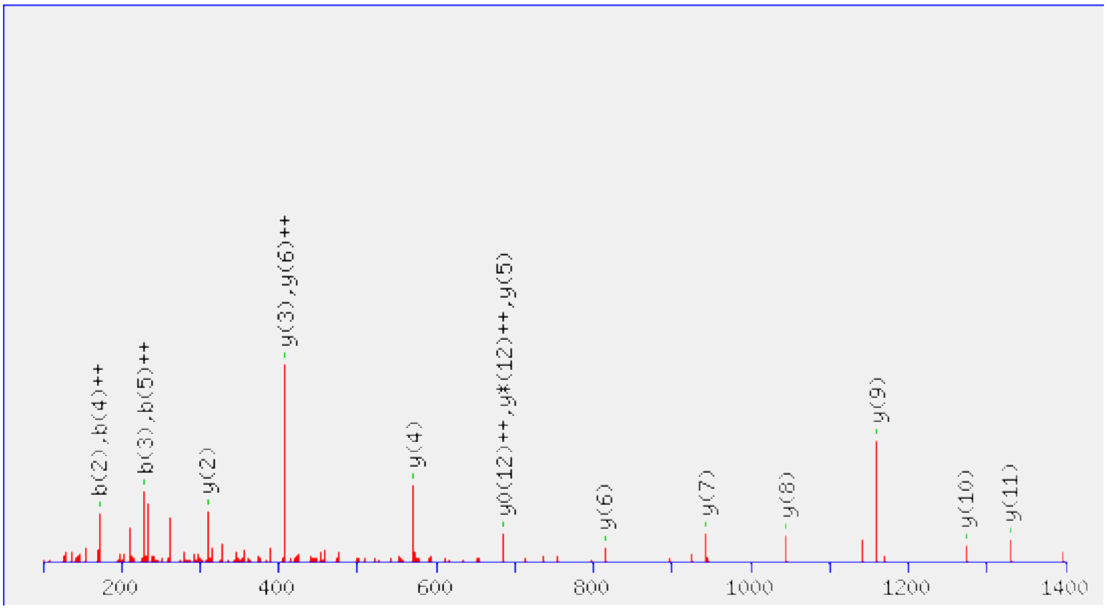

Monoisotopic mass of neutral peptide Mr(calc): 1499.6416
Fixed modifications: Carbamidomethyl (C) (apply to specified residues or termini only)
Ions Score: 105 **Expect:** 6e-08
Matches : 17/128 fragment ions using 13 most intense peaks (help)

Mascot Search Results

Peptide View

Spot no. 5, FIGURE 5B

MS/MS Fragmentation of **TNAENEFVTIK**
Found in **gi|205830697** in **NCBInr**, RecName: Full=Unknown protein 18

Match to Query 125: 1264.654460 from(633.334506,2+) index(124)
Title: File: 130406 3026H.wiff, Sample: 130406 3026H (sample number 1), Elution: 18.454 min, Period: 1, Cycle(s): 3051 (Experiment 3)
Data file F207901.mgf

Click mouse within plot area to zoom in by factor of two about that point

Or, Plot from 100 to 1200 Da Full range

Label all possible matches Label matches used for scoring

Show Y-axis

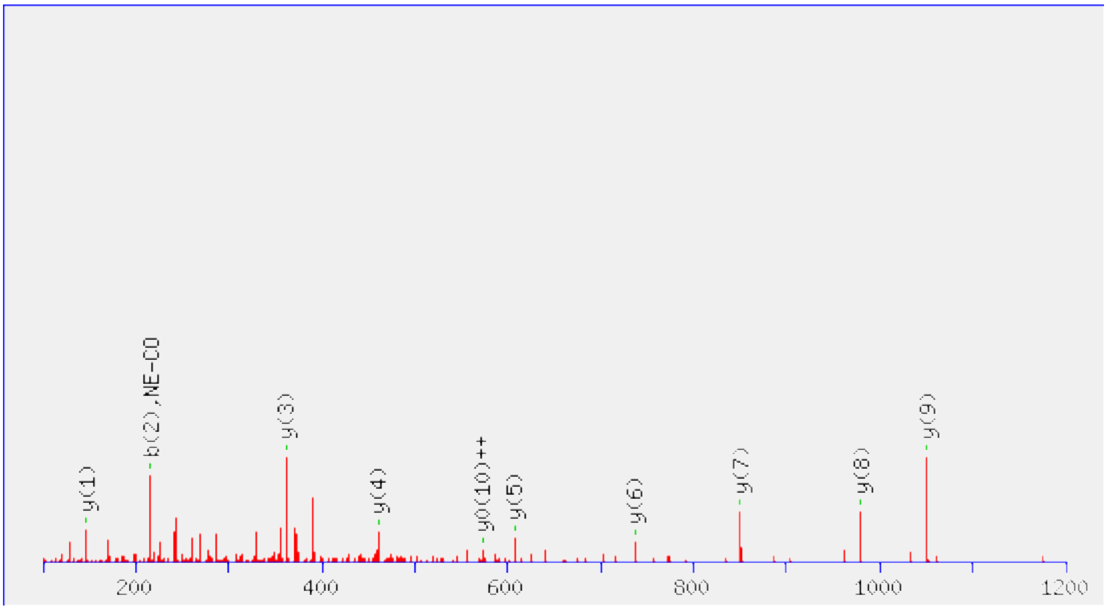

Monoisotopic mass of neutral peptide Mr(calc): 1264.6299
Fixed modifications: Carbamidomethyl (C) (apply to specified residues or termini only)
Ions Score: 80 **Expect:** 6.5e-05
Matches : 12/182 fragment ions using 11 most intense peaks (help)

MATRIX SCIENCE Mascot Search Results

Peptide View **Spot no. 7, FIGURE 5B**

MS/MS Fragmentation of **MEVATDEDFTPIK**

Found in **gi|15242465** **15242465 FREE** in **NCBInr**, soluble inorganic pyrophosphatase 1 [Arabidopsis thaliana]

Match to Query 215: 1510.727682 from(756.371117,2+) index(214)
Title: File: 130406 3026J.wiff, Sample: 130406 3026J (sample number 1), Elution: 18.62 min, Period: 1, Cycle(s): 2871 (Experiment 4)
Data file F207892.mgf

Click mouse within plot area to zoom in by factor of two about that point

Or, Plot from 100 to 1400 Da Full range

Label all possible matches Label matches used for scoring

Show Y-axis

Error: try setting browser cache to automatic.

Monoisotopic mass of neutral peptide Mr(calc): 1510.6861
Fixed modifications: Carbamidomethyl (C) (apply to specified residues or termini only)
Variable modifications:
M1 : Oxidation (M), with neutral losses 0.0000(shown in table), 63.9983
Ions Score: 90 **Expect:** 1.7e-06
Matches : 19/158 fragment ions using 32 most intense peaks (help)

Mascot Search Results

Peptide View

Spot no. 8, FIGURE 5B

MS/MS Fragmentation of **TEETPAVVEEEK**
Found in **gi|9049359** in **NCBInr**, vacuolar calcium binding protein [Raphanus sativus]

Match to Query 210: 1359.672444 from(680.843498,2+) index(209)
Title: File: 130406 3026F.wiff, Sample: 130406 3026F (sample number 1), Elution: 15.577 min, Period: 1, Cycle(s): 2728 (Experiment 2)
Data file F207898.mgf

Click mouse within plot area to zoom in by factor of two about that point
Or, Plot from 100 to 1200 Da Full range
Label all possible matches Label matches used for scoring
Show Y-axis

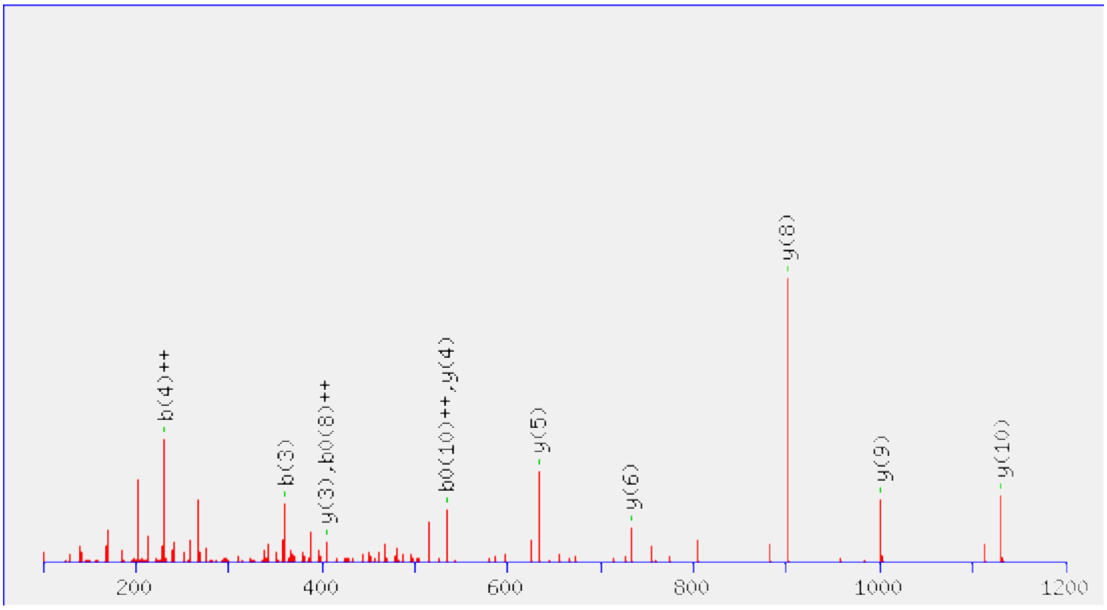

Monoisotopic mass of neutral peptide Mr(calc): 1359.6405
Fixed modifications: Carbamidomethyl (C) (apply to specified residues or termini only)
Ions Score: 61 **Expect:** 0.0015
Matches : 12/108 fragment ions using 11 most intense peaks (help)

Mascot Search Results

Peptide View

Spot no. 9, FIGURE 5B

MS/MS Fragmentation of **TNAENEFVTIKK**
Found in **gi|205830697** in **NCBInr**, RecName: Full=Unknown protein 18

Match to Query 147: 1392.719154 from(465.246994,3+) index(146)
Title: File: 130406 3026D.wiff, Sample: 130406 3026D (sample number 1), Elution: 16.689 to 16.89 min, Period: 1, Cycle(s): 3128 (Experiment 2), 3139 (Experiment 3)
Data file F207896.mgf

Click mouse within plot area to zoom in by factor of two about that point

Or, Plot from 100 to 1200 Da Full range

Label all possible matches ◯ Label matches used for scoring ◉

Show Y-axis ☐

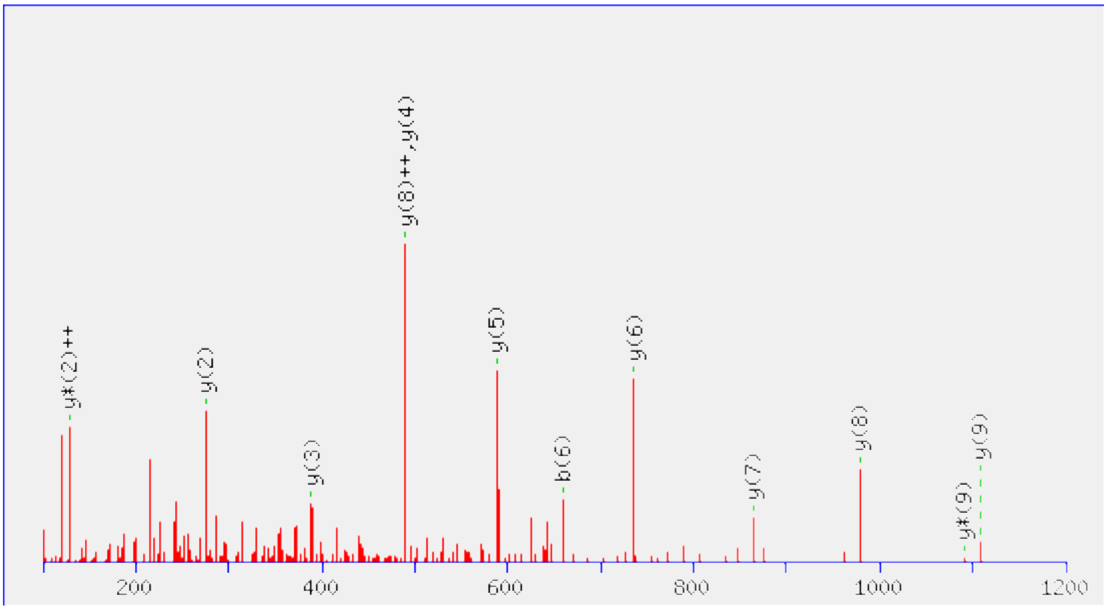

Monoisotopic mass of neutral peptide Mr(calc): 1392.7249
Fixed modifications: Carbamidomethyl (C) (apply to specified residues or termini only)
Ions Score: 78 **Expect:** 3.4e-05
Matches : 12/124 fragment ions using 11 most intense peaks (help)

MATRIX SCIENCE Mascot Search Results

Peptide View **Spot no. 11, FIGURE 5B**

MS/MS Fragmentation of **RPDYIK**
Found in **gi|166700** in **NCBInr**, Fe-superoxide dismutase, partial [Arabidopsis thaliana]

Match to Query 26: 790.525448 from(396.270000,2+) intensity(1705928.0000) index(0)
Title: Cmpd 1, +MSn(396.4), 19.4 min
Data file F207891.mgf

Click mouse within plot area to zoom in by factor of two about that point

Or, Plot from 100 to 750 Da Full range

Label all possible matches Label matches used for scoring

Show Y-axis

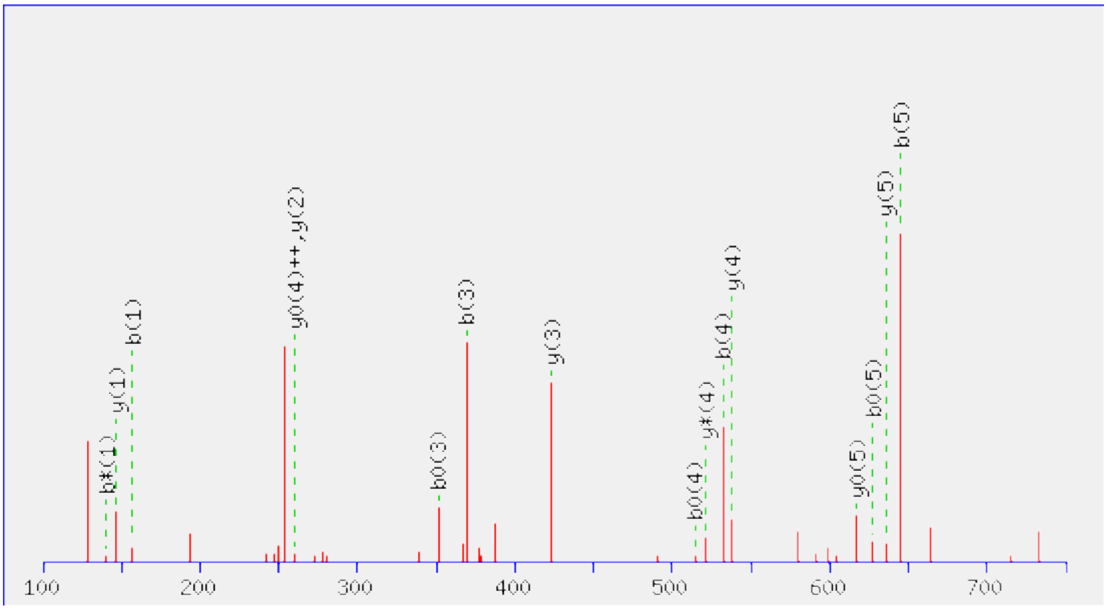

Monoisotopic mass of neutral peptide Mr(calc): 790.4337
Fixed modifications: Carbamidomethyl (C) (apply to specified residues or termini only)
Ions Score: 28 **Expect:** 5.3
Matches : 16/50 fragment ions using 31 most intense peaks (help)

MATRIX SCIENCE

Mascot Search Results

Peptide View **Spot no. 12, FIGURE 5B**

MS/MS Fragmentation of **LDSIGLENTEANR**
Found in **gi|4539316** in **NCBInr**, putative fructose-bisphosphate aldolase [Arabidopsis thaliana]

Match to Query 438: 1430.710234 from(716.362393,2+) index(437)
Title: File: 130406 3026C.wiff, Sample: 130406 3026C (sample number 1), Elution: 17.206 to 17.381 min, Period: 1, Cycle(s): 2684 (Experiment 6), 2688 (Experiment 7)
Data file F207895.mgf

Click mouse within plot area to zoom in by factor of two about that point

Or, Plot from 100 to 1400 Da Full range

Label all possible matches ○ Label matches used for scoring ◉

Show Y-axis ☐

Error: try setting browser cache to automatic.

Monoisotopic mass of neutral peptide Mr(calc): 1430.7001
Fixed modifications: Carbamidomethyl (C) (apply to specified residues or termini only)
Ions Score: 94 **Expect:** 9.3e-07
Matches : 19/122 fragment ions using 32 most intense peaks (help)

Mascot Search Results

Peptide View **Spot no. 14, FIGURE 5B**

MS/MS Fragmentation of **LDSIGLENTEANR**
Found in **gi|18420348** in **NCBInr**, fructose-bisphosphate aldolase, class I [Arabidopsis thaliana]

Match to Query 112: 1430.706752 from(716.360652,2+) index(111)
Title: File: 130406 3026B.wiff, Sample: 130406 3026B (sample number 1), Elution: 17.333 to 17.873 min, Period: 1, Cycle(s): 3219, 3223 (Experiment 2), 3213, 3233 (Experiment 3)
Data file F207894.mgf

Click mouse within plot area to zoom in by factor of two about that point

Or, Plot from 100 to 1400 Da Full range

Label all possible matches Label matches used for scoring

Show Y-axis

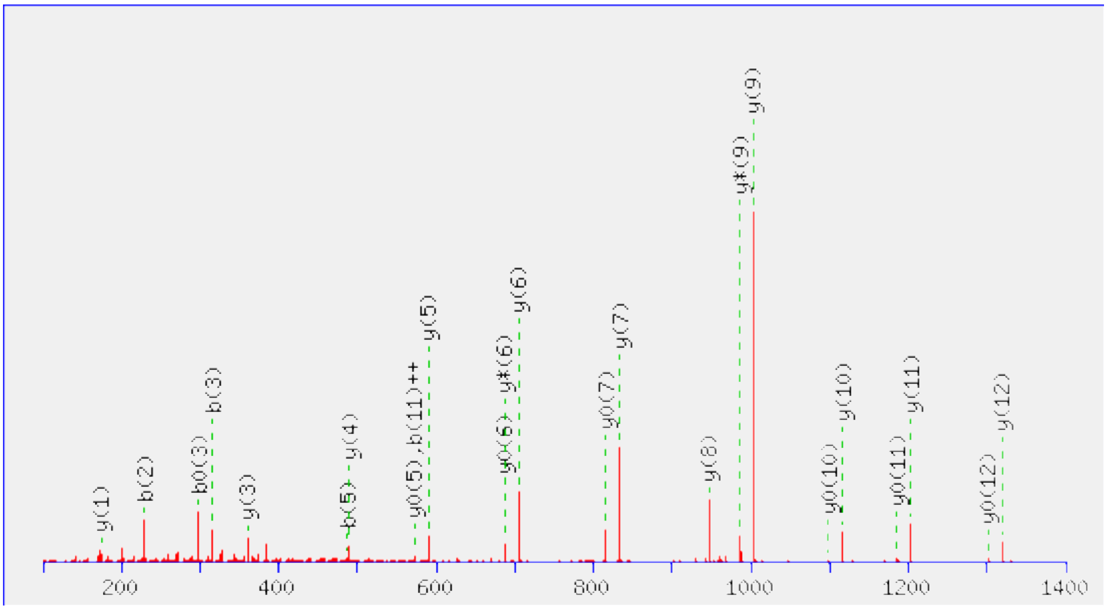

Monoisotopic mass of neutral peptide Mr(calc): 1430.7001
Fixed modifications: Carbamidomethyl (C) (apply to specified residues or termini only)
Ions Score: 116 **Expect:** 5.1e-09
Matches : 24/122 fragment ions using 26 most intense peaks (help)

MATRIX SCIENCE Mascot Search Results

Peptide View **Spot no. 15, FIGURE 5B**

MS/MS Fragmentation of **LTGVTGGDQVAAAMGIYGPR**
Found in **gi|297816906** in **NCBInr**, sedoheptulose-bisphosphatase [Arabidopsis lyrata subsp. lyrata]

Match to Query 103: 1948.988156 from(975.501354,2+) index(102)
Title: File: 130406 3026A.wiff, Sample: 130406 3026A (sample number 1), Elution: 19.553 to 19.598 min, Period: 1, Cycle(s): 3454-3455 (Experiment 3)
Data file F207893.mgf

Click mouse within plot area to zoom in by factor of two about that point
Or, Plot from 100 to 1700 Da Full range
Label all possible matches ○ Label matches used for scoring ◉
Show Y-axis ☐

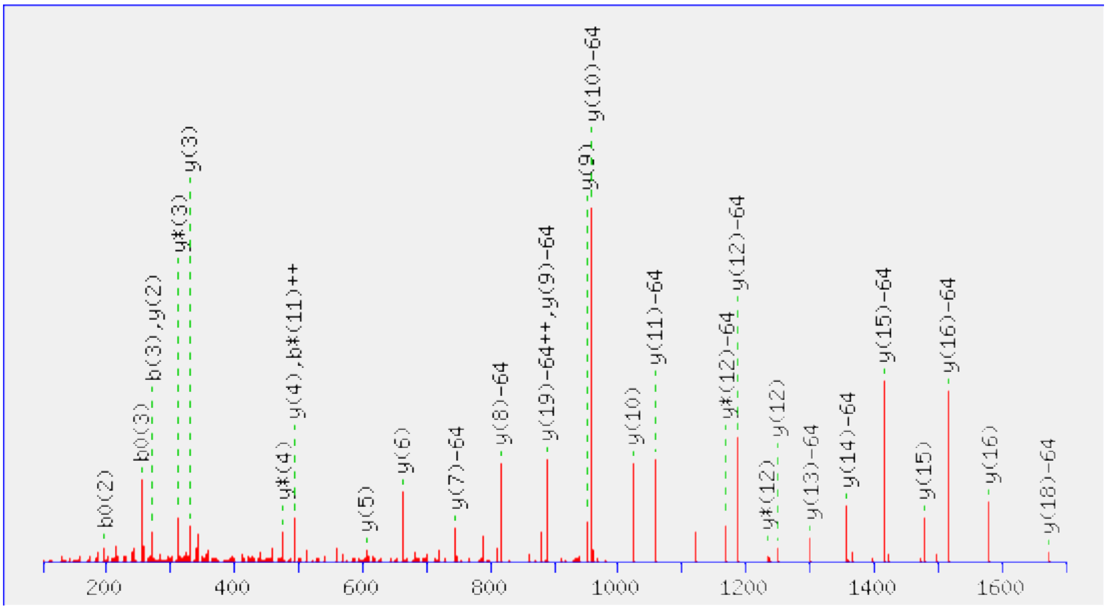

Monoisotopic mass of neutral peptide Mr(calc): 1948.9677
Fixed modifications: Carbamidomethyl (C) (apply to specified residues or termini only)
Variable modifications:
M14 : Oxidation (M), with neutral losses 63.9983(shown in table), 0.0000
Ions Score: 134 **Expect:** 2.3e-10
Matches : 30/288 fragment ions using 31 most intense peaks (help)

Mascot Search Results

Peptide View **SUPPLEMENTARY MATERIAL S1, Band no. 3**

MS/MS Fragmentation of **SPLLLQSNPIHK**
Found in **gi|87294807** in **NCBInr**, glutathione S-transferase [Brassica napus]

Match to Query 166: 1346.172724 from(674.093638,2+) index(248)
Title: RC-1-JASS-190309_090319140534.1796.1798.2.dta
Data file RC-1-JASS merge.mgf

Click mouse within plot area to zoom in by factor of two about that point

Or, Plot from 100 to 1300 Da Full range

Label all possible matches Label matches used for scoring

Show Y-axis

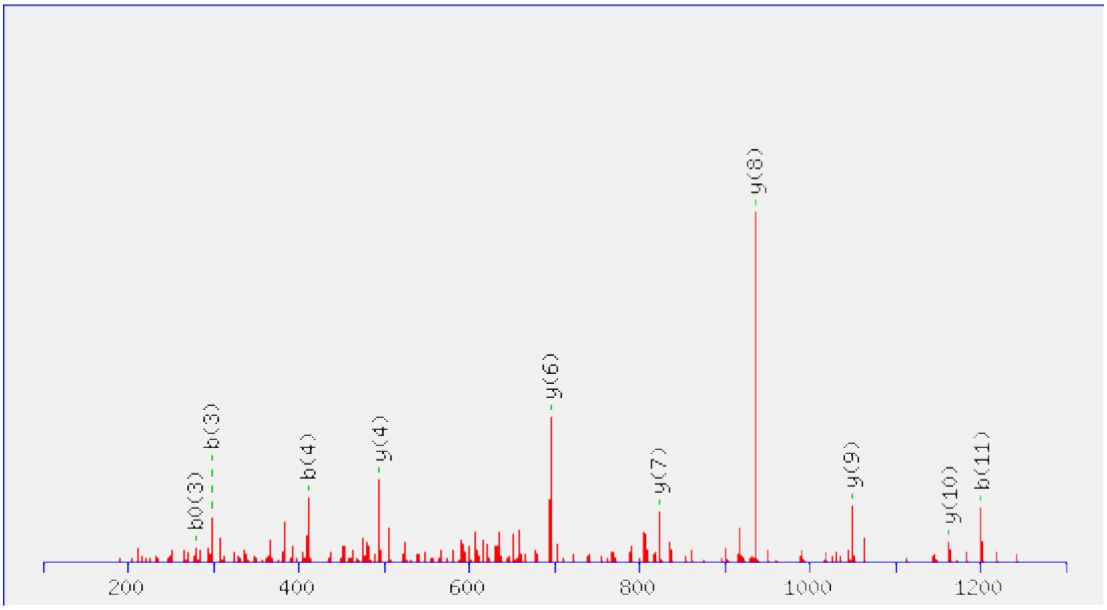

Monoisotopic mass of neutral peptide Mr(calc): 1345.7718
Fixed modifications: Carbamidomethyl (C) (apply to specified residues or termini only)
Ions Score: 52 **Expect:** 0.019
Matches : 10/112 fragment ions using 11 most intense peaks

Mascot Search Results

Peptide View **SUPPLEMENTARY MATERIAL S1, Band no. 3**

MS/MS Fragmentation of **YAGTEVEFNDVK**
Found in **gi|3057150** in **NCBInr**, chaperonin 10 [Arabidopsis thaliana]

Match to Query 178: 1371.282724 from(686.648638,2+) index(206)
Title: RC-1-JASS-190309_090319140534.1726.1726.2.dta
Data file RC-1-JASS merge.mgf

Click mouse within plot area to zoom in by factor of two about that point

Or, Plot from 200 to 1300 Da Full range

Label all possible matches Label matches used for scoring

Show Y-axis

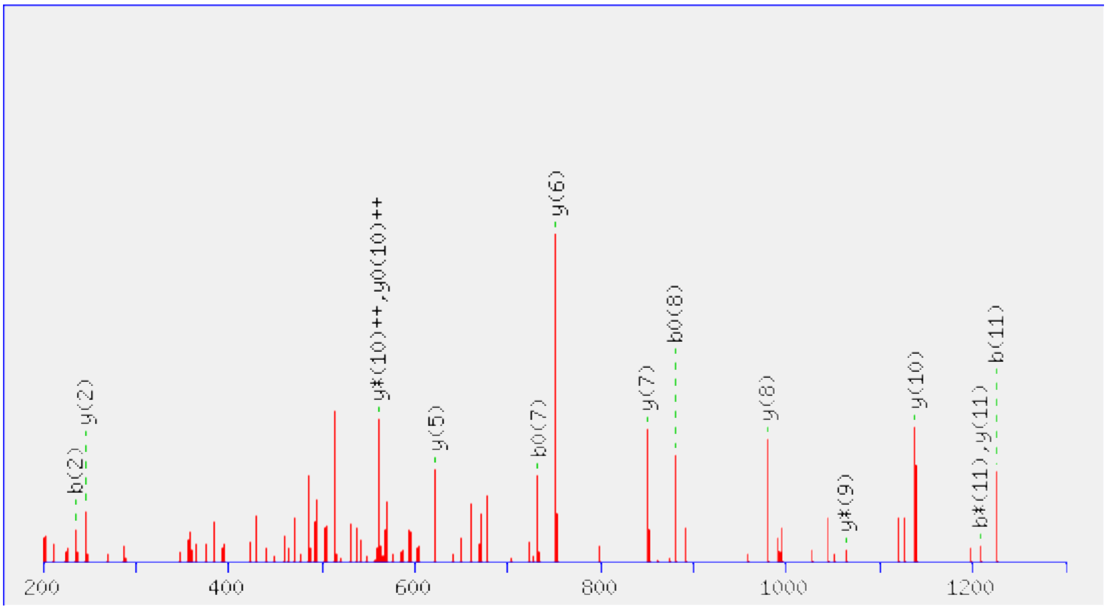

Monoisotopic mass of neutral peptide Mr(calc): 1370.6354
Fixed modifications: Carbamidomethyl (C) (apply to specified residues or termini only)
Ions Score: 49 **Expect:** 0.045
Matches : 15/106 fragment ions using 22 most intense peaks (help)

Mascot Search Results

Peptide View SUPPLEMENTARY MATERIAL S1, Band no. 3

MS/MS Fragmentation of **VISELGDSAFEDQCGR**
Found in **gi|297787439** in **NCBInr**, chloroplast beta-carbonic anhydrase [Brassica napus]

Match to Query 325: 1782.312724 from(892.163638,2+) index(259)
Title: RC-1-JASS-190309_090319140534.1820.1822.2.dta
Data file RC-1-JASS merge.mgf

Click mouse within plot area to zoom in by factor of two about that point
Or, Plot from 200 to 1700 Da Full range
Label all possible matches Label matches used for scoring
Show Y-axis

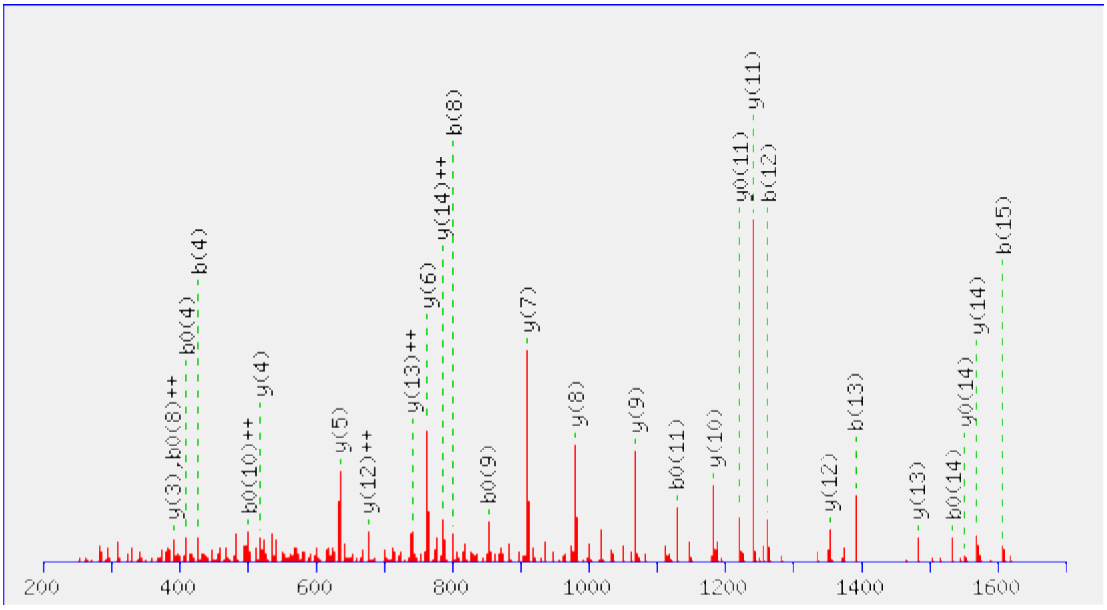

Monoisotopic mass of neutral peptide Mr(calc): 1781.7890
Fixed modifications: Carbamidomethyl (C) (apply to specified residues or termini only)
Ions Score: 82 **Expect:** 1.9e-05
Matches : 28/144 fragment ions using 56 most intense peaks (help)

{MATRIX SCIENCE} Mascot Search Results

Peptide View SUPPLEMENTARY MATERIAL S1, Band no. 6

MS/MS Fragmentation of **FITKLDEEGGPEAR**
Found in **gi|211905345** in **NCBInr**, epithiospecifier protein [Brassica rapa subsp. pekinensis]

Match to Query 233: 1560.282724 from(781.148638,2+) index(224)
Title: RC-5-JAS-DU-190309.1622.1622.2.dta
Data file RC-5 JAS merge.mgf

Click mouse within plot area to zoom in by factor of two about that point

Or, Plot from 200 to 1500 Da Full range

Label all possible matches Label matches used for scoring

Show Y-axis

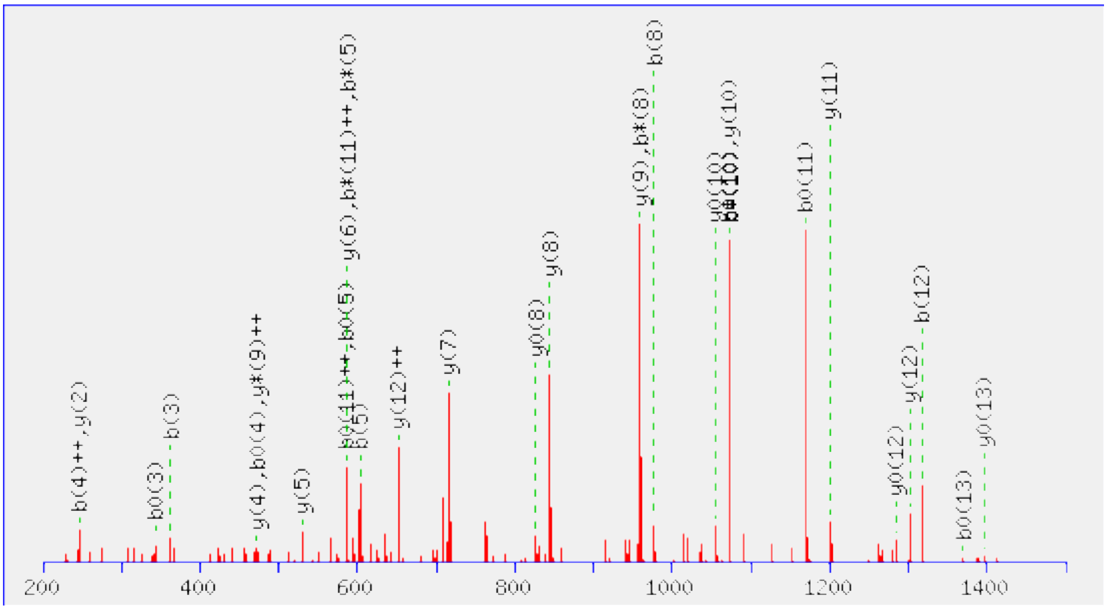

Monoisotopic mass of neutral peptide Mr(calc): 1560.7784
Fixed modifications: Carbamidomethyl (C) (apply to specified residues or termini only)
Ions Score: 74 **Expect:** 0.00013
Matches : 32/142 fragment ions using 36 most intense peaks (help)

Mascot Search Results

Peptide View SUPPLEMENTARY MATERIAL S1, Band no. 6

MS/MS Fragmentation of **LDSIGLENTEANR**
Found in **gi|16226653** in **NCBInr**, AT4g38970/F19H22_70 [Arabidopsis thaliana]

Match to Query 185: 1430.832724 from(716.423638,2+) index(237)
Title: RC-5-JAS-DU-190309.1642.1644.2.dta
Data file RC-5 JAS merge.mgf

Click mouse within plot area to zoom in by factor of two about that point

Or, Plot from 200 to 1400 Da Full range

Label all possible matches Label matches used for scoring

Show Y-axis

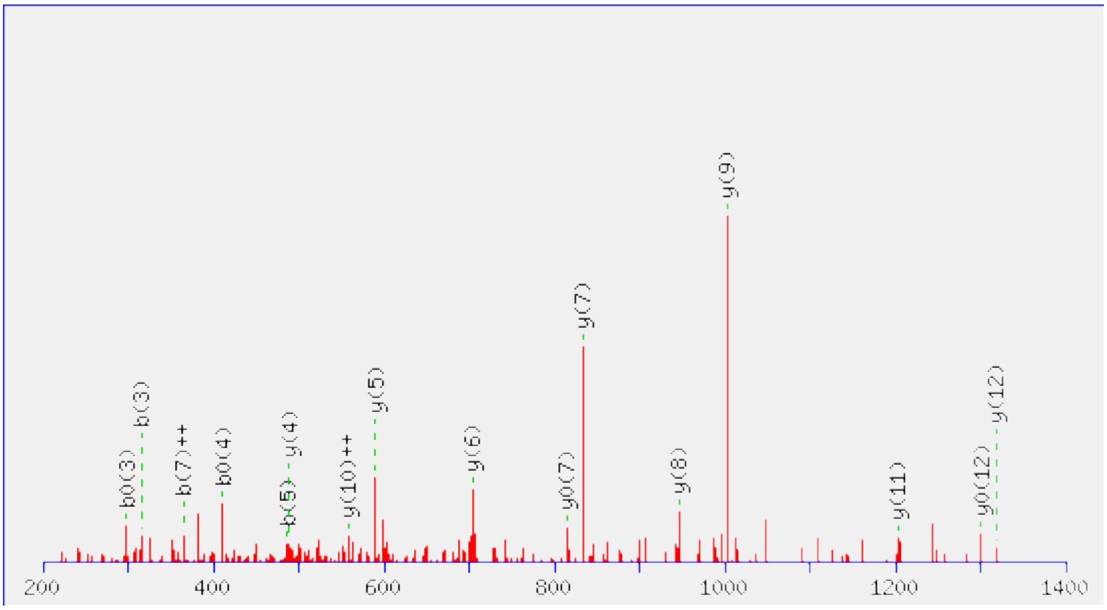

Monoisotopic mass of neutral peptide Mr(calc): 1430.7001
Fixed modifications: Carbamidomethyl (C) (apply to specified residues or termini only)
Ions Score: 47 **Expect:** 0.12
Matches : 16/122 fragment ions using 33 most intense peaks (help)

Mascot Search Results

Peptide View SUPPLEMENTARY MATERIAL S1, Band no. 6

MS/MS Fragmentation of **VPTVDVSVVDLTVR**
Found in **gi|120675** in **NCBInr**, RecName: Full=Glyceraldehyde-3-phosphate dehydrogenase, cytosolic

Match to Query 208: 1497.912724 from(749.963638,2+) index(439)
Title: RC-5-JAS-DU-190309.2033.2033.2.dta
Data file RC-5 JAS merge.mgf

Click mouse within plot area to zoom in by factor of two about that point

Or, [Plot from] [200] to [1500] Da [Full range]

Label all possible matches ○ Label matches used for scoring ◉

Show Y-axis ☐

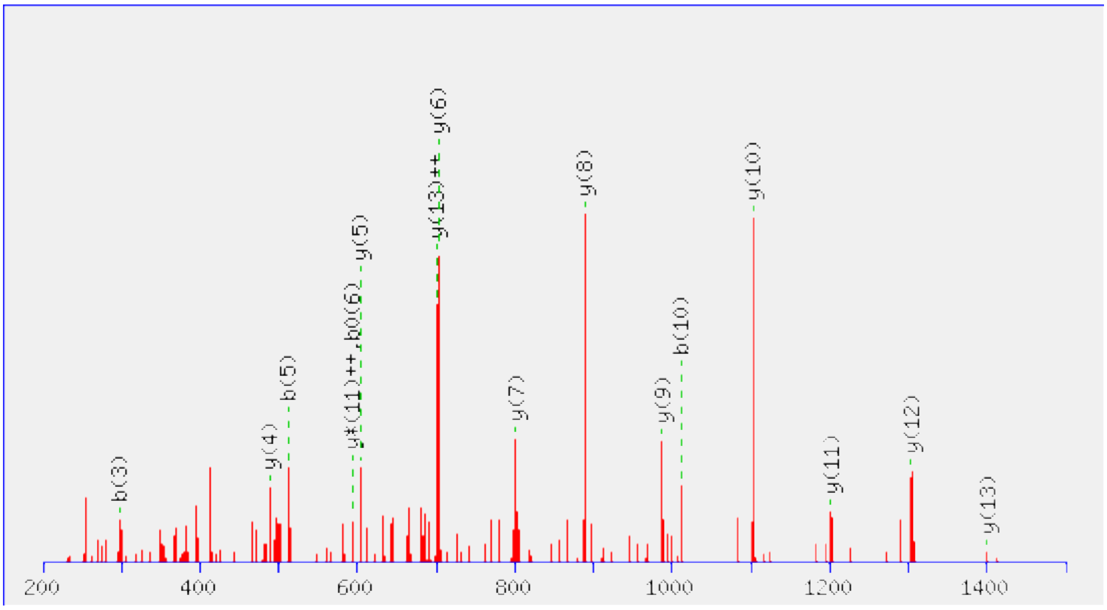

Monoisotopic mass of neutral peptide Mr(calc): 1497.8403
Fixed modifications: Carbamidomethyl (C) (apply to specified residues or termini only)
Ions Score: 75 **Expect:** 0.00015
Matches : 16/122 fragment ions using 24 most intense peaks (help)

Mascot Search Results

Peptide View SUPPLEMENTARY MATERIAL S1, Band no. 6

MS/MS Fragmentation of **VPTVDVSVVDLTVR**
Found in **gi|312282755** in **NCBInr**, unnamed protein product [Thellungiella halophila]

Match to Query 208: 1497.912724 from(749.963638,2+) index(439)
Title: RC-5-JAS-DU-190309.2033.2033.2.dta
Data file RC-5 JAS merge.mgf

Click mouse within plot area to zoom in by factor of two about that point

Or, Plot from 200 to 1500 Da Full range

Label all possible matches Label matches used for scoring

Show Y-axis

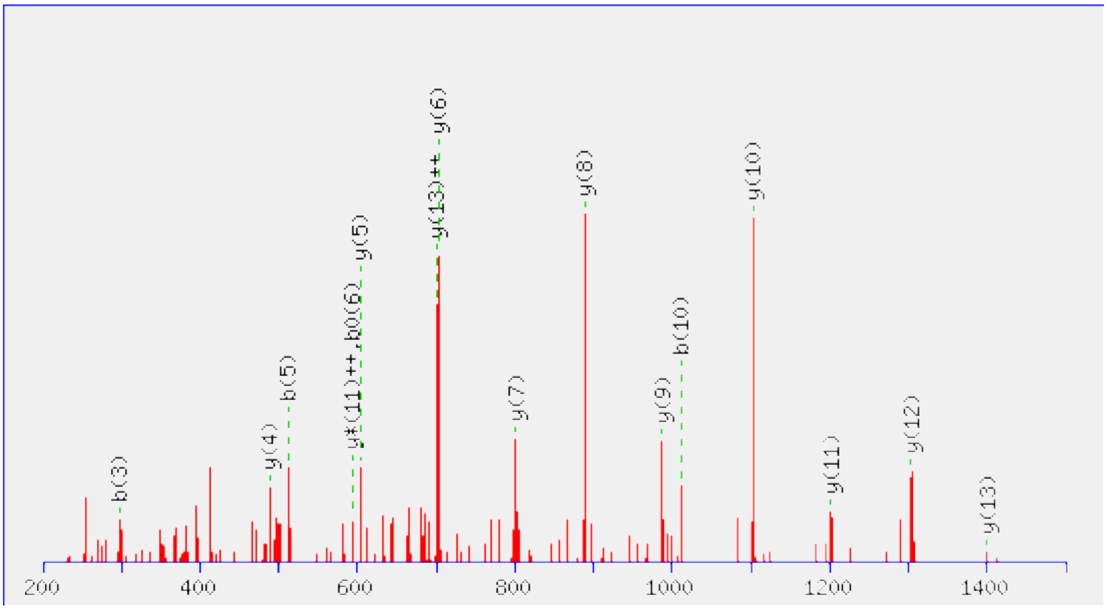

Monoisotopic mass of neutral peptide Mr(calc): 1497.8403
Fixed modifications: Carbamidomethyl (C) (apply to specified residues or termini only)
Ions Score: 75 **Expect:** 0.00015
Matches : 16/122 fragment ions using 24 most intense peaks (help)

MATRIX SCIENCE Mascot Search Results

Peptide View **SUPPLEMENTARY MATERIAL S1, Band no. 7**

MS/MS Fragmentation of **GIFTNVTSPTAK**
Found in **gi|297816906** in **NCBInr**, sedoheptulose-bisphosphatase [Arabidopsis lyrata subsp. lyrata]

Match to Query 89: 1234.745448 from(618.380000,2+) intensity(4842240.0000) index(9)
Title: Cmpd 10, +MSn(618.6), 26.1 min
Data file JAS RC 1merge.mgf

Click mouse within plot area to zoom in by factor of two about that point
Or, Plot from 200 to 1100 Da Full range
Label all possible matches Label matches used for scoring
Show Y-axis

Error: try setting browser cache to automatic.

Monoisotopic mass of neutral peptide Mr(calc): 1234.6558
Fixed modifications: Carbamidomethyl (C) (apply to specified residues or termini only)
Ions Score: 70 **Expect:** 0.00065
Matches : 20/114 fragment ions using 31 most intense peaks (help)

Mascot Search Results

Peptide View **SUPPLEMENTARY MATERIAL S1, Band no. 8**

MS/MS Fragmentation of **GLEGHVMEQLK**
Found in **gi|18379240** in **NCBInr**, MLP-like protein 328 [Arabidopsis thaliana]

Match to Query 224: 1239.752724 from(620.883638,2+) index(141)
Title: RC-3-JAS-DU-190309.1671.1673.2.dta
Data file RC-3JASS merge.mgf

Click mouse within plot area to zoom in by factor of two about that point

Or, Plot from 150 to 1150 Da Full range

Label all possible matches Label matches used for scoring

Show Y-axis

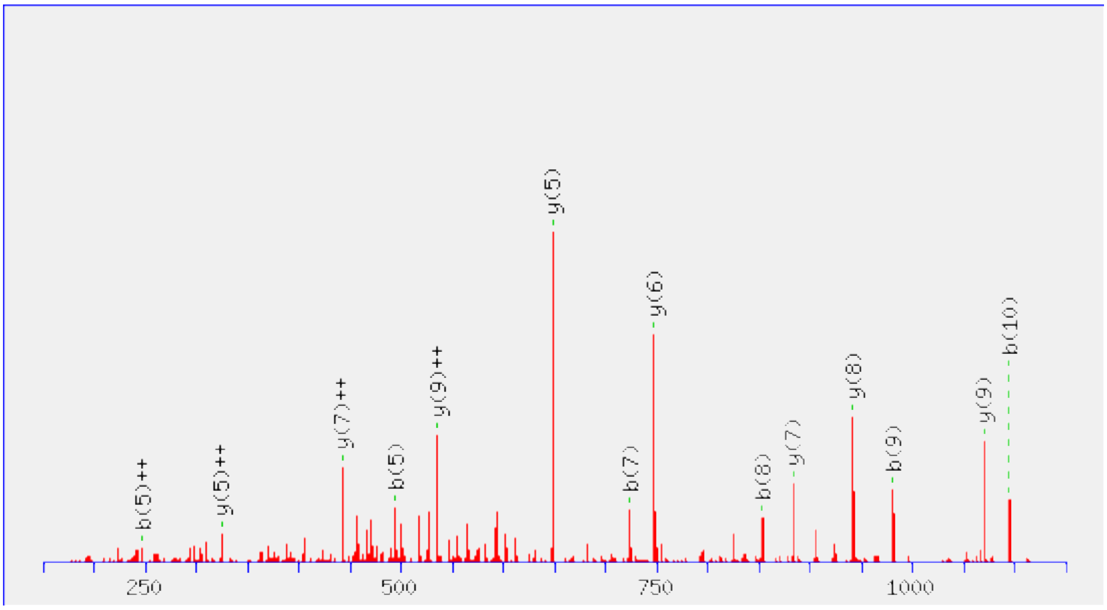

Monoisotopic mass of neutral peptide Mr(calc): 1239.6281
Fixed modifications: Carbamidomethyl (C) (apply to specified residues or termini only)
Ions Score: 47 **Expect:** 0.099
Matches : 14/94 fragment ions using 20 most intense peaks (help)

Mascot Search Results

Peptide View SUPPLEMENTARY MATERIAL S1, Band no. 9

MS/MS Fragmentation of **FVEDTASSFSVA**
Found in **gi|131390** in **NCBInr**, RecName: Full=Oxygen-evolving enhancer protein 2, chloroplastic; Short=OEE2; AltName: Full=23 kDa subunit of oxygen evolving system of photosystem II; AltName: Full=23 kDa thylakoid membrane protein; AltName: Full=OEC 23 kDa subunit; Flags: Precurso

Match to Query 172: 1258.565448 from(630.290000,2+) intensity(304450.0000) index(63)
Title: Cmpd 64, +MSn(630.8), 29.7 min
Data file DU-JASMEET-RC-7.mgf

Click mouse within plot area to zoom in by factor of two about that point

Or, Plot from 200 to 1100 Da Full range

Label all possible matches Label matches used for scoring

Show Y-axis

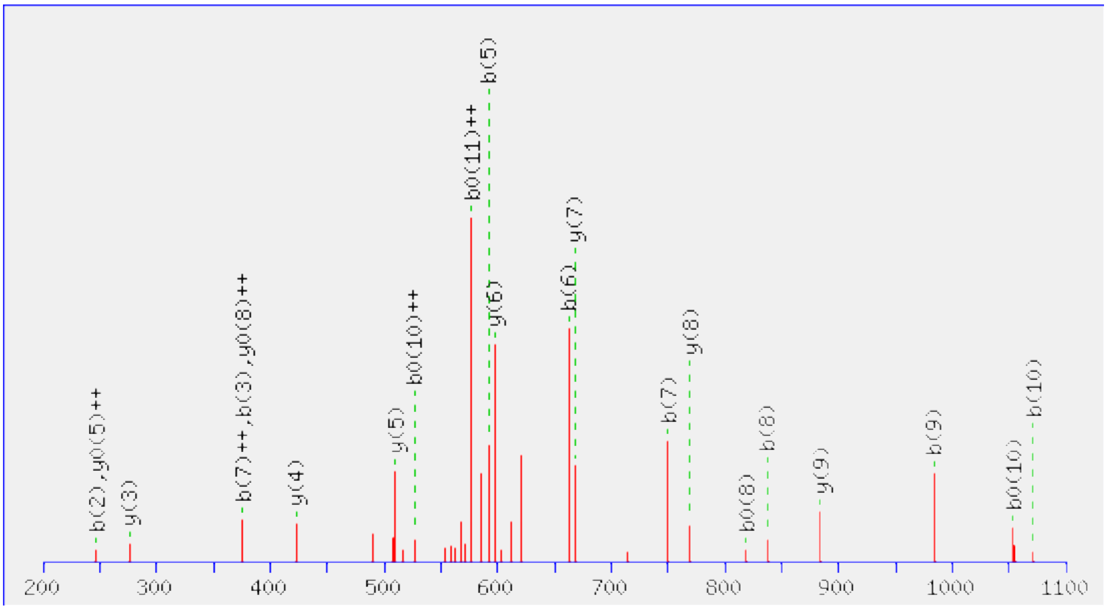

Monoisotopic mass of neutral peptide Mr(calc): 1258.5718
Fixed modifications: Carbamidomethyl (C) (apply to specified residues or termini only)
Ions Score: 76 **Expect:** 0.00015
Matches : 22/80 fragment ions using 24 most intense peaks

Mascot Search Results

Peptide View **SUPPLEMENTARY MATERIAL S1, Band no. 9**

MS/MS Fragmentation of **HQLITATVSDGK**
Found in **gi|242047384** in **NCBInr**, hypothetical protein SORBIDRAFT_02g002690 [Sorghum bicolor]

Match to Query 178: 1268.465448 from(635.240000,2+) intensity(270343.0000) index(2)
Title: Cmpd 3, +MSn(635.8), 22.5 min
Data file DU-JASMEET-RC-7.mgf

Click mouse within plot area to zoom in by factor of two about that point
Or, Plot from 200 to 1150 Da Full range
Label all possible matches Label matches used for scoring
Show Y-axis

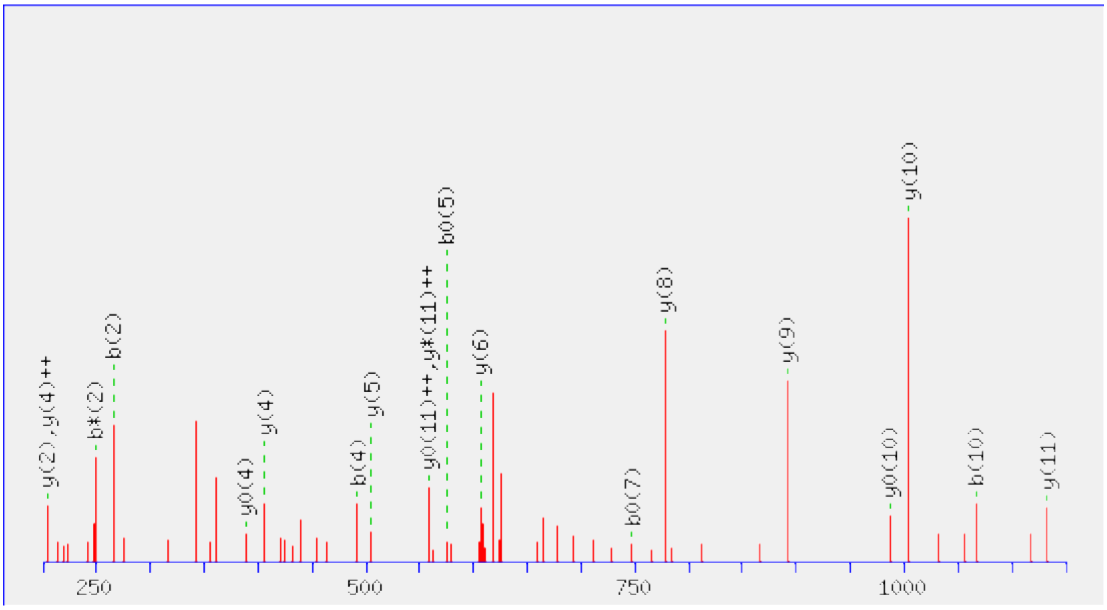

Monoisotopic mass of neutral peptide Mr(calc): 1268.6725
Fixed modifications: Carbamidomethyl (C) (apply to specified residues or termini only)
Ions Score: 63 **Expect:** 0.0023
Matches : 19/118 fragment ions using 27 most intense peaks (help)
