# Supplementary material for: RuBisCO depletion improved proteome coverage of cold responsive S-nitrosylated targets in Brassica juncea
Source: Front Plant Sci. 2013 Sep 2;4:342. doi: 10.3389/fpls.2013.00342 (PMC3759006; doi:10.3389/fpls.2013.00342)
Supplement: Supplementary file 2 [file DataSheet2.PDF]

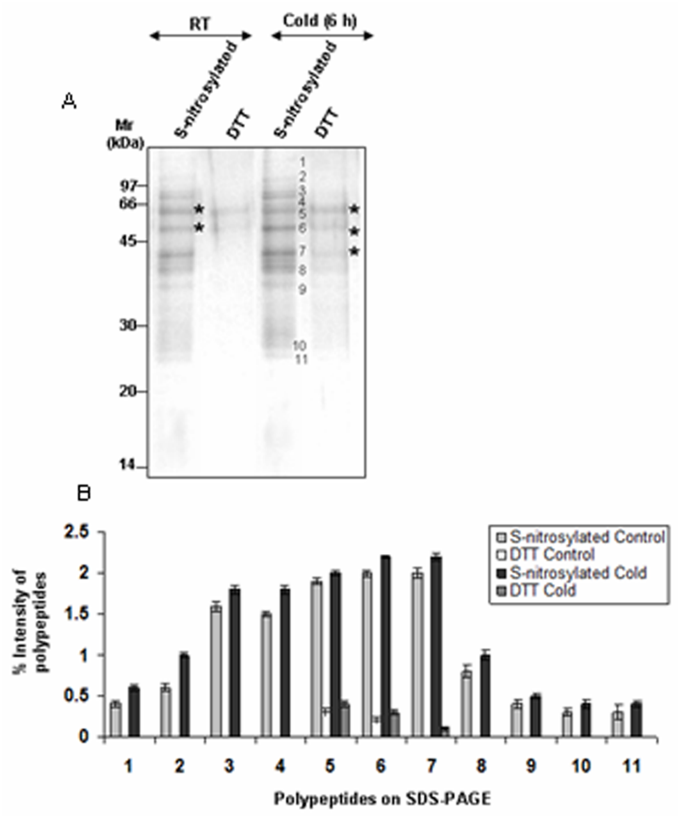

Supplementary material S1. (A) Silver stained 12% SDS-PAGE of cold (6 h) stress modulated S-nitrosylated proteins and their comparison with the control. Numbers marked next to the polypeptide indicate S-nitrosylated proteins. (B) Bar diagram showing relative quantification of the polypeptides of SDS-PAGE gel. The intensity of the polypeptide was quantified using densitometric scanning of AlphaImager software (Alpha Innotech Corporation). Polypeptide intensities were calculated by subtracting background intensity. The results are representative of three biological replicates.

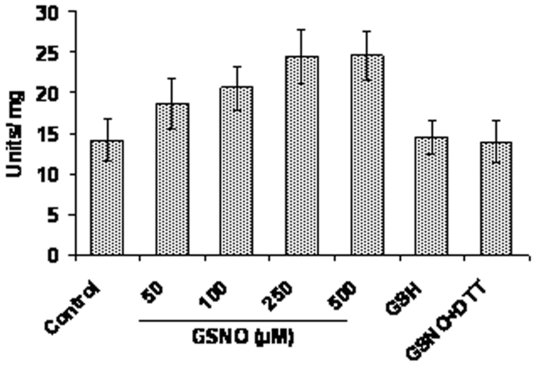

Supplementary material S2. Effect of S-nitrosylation on Fructose bisphosphate aldolase activity. *B. juncea* seedling extracts were either untreated (control) or treated with GSNO or GSH (as indicted) prior to the measurement of enzyme activity. GSNO (250 µM) treated extract was also treated with 10 mM DTT, which restored the enzyme activity.
